# Hunter-Gatherers in context: Mammal community composition in a northern Tanzania landscape used by Hadza foragers and Datoga pastoralists

**DOI:** 10.1371/journal.pone.0251076

**Published:** 2021-05-14

**Authors:** Brian M. Wood, Riccardo S. Millar, Nicholas Wright, Joshua Baumgartner, Hannah Holmquist, Christian Kiffner

**Affiliations:** 1 Department of Human Behavior, Ecology and Culture, Max Planck Institute for Evolutionary Anthropology, Leipzig, Germany; 2 Department of Anthropology, University of California, Los Angeles, CA, United States of America; 3 Dickinson College, Carlisle, PA, United States of America; 4 Vassar College, Poughkeepsie, NY, United States of America; 5 University of Wisconsin-Madison, Madison, WI, United States of America; 6 Iowa State University, Ames, IA, United States of America; 7 Center For Wildlife Management Studies, The School For Field Studies, Karatu, Tanzania; Universita degli Studi di Firenze Dipartimento di Biologia, ITALY

## Abstract

In many regions of sub Saharan Africa large mammals occur in human-dominated areas, yet their community composition and abundance have rarely been described in areas occupied by traditional hunter-gatherers and pastoralists. Surveys of mammal populations in such areas provide important measures of biodiversity and provide ecological context for understanding hunting practices. Using a sampling grid centered on a Hadza hunter-gatherer camp and covering 36 km^2^ of semi-arid savannah in northern Tanzania, we assessed mammals using camera traps (n = 19 stations) for c. 5 months (2,182 trap nights). In the study area (*Tli’ika* in the Hadza language), we recorded 36 wild mammal species. Rarefaction curves suggest that sampling effort was sufficient to capture mammal species richness, yet some species known to occur at low densities in the wider area (e.g. African lions, wildebeest) were not detected. Relative abundance indices of wildlife species varied by c. three orders of magnitude, from a mean of 0.04 (African wild dog) to 20.34 capture events per 100 trap-nights (Kirk’s dik dik). To contextualize the relative abundance of wildlife in the study area, we compared our study’s data to comparable camera trap data collected in a fully protected area of northern Tanzania with similar rainfall (Lake Manyara National Park). Raw data and negative binomial regression analyses show that wild herbivores and wild carnivores were generally detected in the national park at higher rates than in the Hadza-occupied region. Livestock were notably absent from the national park, but were detected at high levels in Tli’ika, and cattle was the second most frequently detected species in the Hadza-used area. We discuss how these data inform current conservation efforts, studies of Hadza hunting, and models of hunter-gatherer foraging ecology and diet.

## Introduction

Across Africa, populations of many large mammal species have been declining inside and outside of protected areas [1–5] but most of our understanding of the processes generating population declines of mammal species has focused on species within protected areas [5–11]. While the causes of population declines and local extinctions vary, there is a general consensus among conservationists that ‘fortress conservation’ efforts striving towards full protection from human exploitation are not sufficient to ensure the long term survival of many mammal species [12–16]. Consequently, wildlife conservation efforts have gradually shifted towards understanding the dynamics of human-wildlife coexistence across more diverse landscapes [17–19]. To assess possibilities for sustained coexistence of people and wildlife, it is especially important to assess wildlife populations in areas that are used by subsistence hunters [20]. Although exploitative by definition, subsistence hunting is considered to be an initial stage of the defaunation process, often of smaller effect than that caused by the use of sophisticated weapons, market-driven hunting, and large-scale land conversion [21]. Unfortunately, however, there is a paucity of mammal monitoring programs in lands used by subsistence hunter-gatherers, which hampers our understanding of these particular coupled human-ecological systems and their conservation implications [22].

In East Africa, scholars have observed widely varying human impacts upon wildlife communities. In areas subject to human land-uses such as dense settlement, agriculture and pastoralism, some communities of large mammals show signs of low species richness and density, while others exhibit species richness and densities similar to nearby protected areas [16,23–32]. Cultural and economic differences among human communities are clearly implicated in such patterns, but poorly understood. In this study, we describe the mammal communities found in the Lake Eyasi/Yaeda Valley region (Tanzania), in an area occupied year-round by Hadza hunter-gatherers and seasonally by Datoga pastoralists. The study area is outside any national park, but it is within a large area of contiguous wildlife habitat southeast of Lake Eyasi that is characterized by generally low human population density and low rates of agricultural land conversion or deforestation. The area is adjacent to areas subject to village-based land use plans that place some limits on exploitative activities, but which are not enforced consistently, owing to a lack of resources. Assessing wildlife communities in such areas is of vital conservation significance. Relative to wildlife research in protected areas, research outside of parks requires greater engagement, support, and guidance by the local people using the shared landscapes. Employing participatory camera-trap monitoring [33], we describe the mammal species detected in the area centered upon a favored camping location of Hadza hunter-gatherers. This study builds upon previous research in which mammal communities across the wider study area were estimated using indirect (animal signs) evidence [32]. The status of wildlife populations is a vital concern to the Hadza community and is valuable for modeling the ecology of hunting and gathering in the region. Knowledge of mammal community composition is also useful for community-based land use planning and conservation in the area, efforts which currently involve collaboration between representatives of the Hadza and Datoga communities, village governments, and conservation groups funded through carbon offsets [34].

Prior studies of subsistence hunters have documented patterns of local depletion of hunted species near settlements, but whether these local impacts translate into lower regional biodiversity depends on a host of factors, including the size and density of hunting communities, the technologies employed, and the scale of market-driven hunting [35,36]. Hadza hunting practices have been described as focused on “immediate returns”, and unlike norms commonly applied in formal wildlife management, do not include restrictions on killing prey based on their age, sex, or number [37]. Traditionally, local groups of Hadza are non-territorial and permit open-access to landscapes used for harvesting foods [37]. The Hadza are also known to prize the killing of large-bodied mammals [38]. Such species are particularly vulnerable to hunting pressures, because of their slow reproductive rates [39]. However, recent detailed research documents the fact that most prey items killed by Hadza are small wildlife, less than 10 kg in body mass [40]. Variable hunting pressures can have substantial direct and indirect effects on the persistence of wildlife populations [36] and the sustainability of hunting practices [41], yet long term monitoring and engagement are needed to assess such effects. Our work here is focused on characterizing the mammal community and contributing to such efforts.

While a substantial increase in livestock populations across East Africa has led to research into the potentially negative effects of livestock on wildlife communities [42,43], research into the direct and indirect effects of livestock upon the livelihoods of subsistence hunters is not well understood. The landscape we monitored is used seasonally by Datoga pastoralists, who migrate into the hilly area of Tli’ika during the dry season (~ June-October). The hunting of large and dangerous animals, particularly lions (*Panthera leo*) and African buffalo (*Syncerus caffer*), is highly prized in Datoga culture as a way for young men to signal their bravery and receive gifts of cattle, but hunting for subsistence purposes is rare [44,45]. While the Datoga’s direct impact upon wildlife populations through hunting is likely to be quite targeted and low overall, the effects of pastoralism on wildlife communities are debated [46–50]. The Hadza themselves adamantly claim that the Datoga’s livestock degrade the land’s carrying capacity for wildlife, and impedes their hunting. The abundance of livestock within the Hadza’s traditional lands is a local political issue and is important for contextualizing Hadza subsistence hunting.

An earlier study proposed that wildlife densities in this study area were equivalent to those observed in national parks with similar rainfall [51]. This comparison was based on aerial wildlife surveys, which were carried out in the region in the 1970s and 1980s. Here, using camera trap detection rates, we provide an updated comparison between the study area and the nearest national park, Lake Manyara National Park (LMNP), which has similar rainfall (~ 500 mm/year), and provides a useful point of reference for discussing mammal abundances in Hadza-occupied areas. If Hadza hunters experienced the high wildlife densities that are observed in national parks of similar rainfall, as conjectured by [52], this would suggest that their comtemporary hunting practices could serve as a referential model for hunters living in environments minimally affected by agricultural and pastoral populations. It is important to note that the claim of high wildlife abundance in the Hadza region put forth in [52] is based on a single aerial survey conducted in 1977. As later pointed out by Blurton Jones [51; pages 30–31], subsequent aerial surveys reveal a strong pattern of declining wildlife populations in the area: 4.45 individuals/km^2^ in 1977, 2.09 in 1989, and 0.87 in 1992. In order to test whether wildlife densities in the core hunting territories of contemporary Hadza are similar to that of LMNP, we compare rates of wild mammal detection in this study to rates recorded in a similar study done recently within Lake Manyara National Park.

If there is evidence for recent and significant declines in wildlife species in the Hadza used landscape, and also large numbers of livestock detected in this region, then the hunting practices of contemporary Hadza are likely to have also changed recently, adapting to these new challenges. This issue is also relevant to a wider debate in anthropology concerning the degree to which ethnographically documented hunter-gatherers occupy habitats that are representantive of past environments used by hunters before the neolithic revolution; perhaps recently studied hunter-gatherers occupy lower quality habitats, namely those less suitable for agriculture and on the whole having lower carrying capacities for forager populations [52–56]. Recent studies have addressed this issue by comparing the net primary productivity (NPP) of forager-occupied habitats to those of farmer-occupied habitats, treating NPP as a proxy measure for general environmental quality. In the discussion, we use our camera-trap results to discuss the limitations of NPP-based measures of habitat quality.

To provide data of value to wildlife conservation efforts and longitudinal anthropological research in this area, we (1) describe mammal species richness detected in our survey, (2) report species-specific relative abundance indices of wildlife and livestock, and (3) compare species-specific relative abundance indices in our survey to those detected using similar methods in Lake Manyara National Park.

## Methods

This field research was permitted by the Tanzanian Wildlife Research Institute (TAWIRI) and the Tanzanian Commission for Science and Technology (COSTECH) (permit #: 2017-288-ER-2013-191) and subsequent explicit consent from local authorities.

### Study area

East of Lake Eyasi, in northern Tanzania, approximately 1000 people speak the Hadza language, and most members of this community continue to hunt and gather for wild foods using traditional technologies. Migration, population growth, and agricultural development near several villages in the region (Mangola, Mitala, Munguli, Yaeda Chini) has drastically altered the landscape that was historically occupied by Hadza [57,58]. Owing to population growth and land conversion, some areas that were important camping and hunting areas in living memory are no longer visited or used by Hadza, and the total extent of areas considered suitable for subsistence hunting has declined [58]. We carried out this research in a geographic area southeast of Lake Eyasi in a sub-region known to the Hadza as Tli’ika, which corresponds to the placename ‘Kideru Hills’ on some maps. Our camera trapping survey area is remote from agricultural villages (approx. 7 km distance as the crow flies through rocky terrain), and relatively unaffected by the migration, population growth, and agricultural development in other areas. This region continues to support the sustained presence of 200–400 Hadza community members who hunt mammals and birds using bows and arrows [57–59]. The surveyed area is also occupied seasonally by Datoga pastoralists, who bring their livestock into Tli’ika during the dry season, after cattle have denuded the grasses of the Yaeda Valley and other lower lying areas around Lake Eyasi.

The area is characterized by woodland savannah habitat, with prominent stands of baobab (*Adansonia digitata*), acacia (*Vachellia* spp.), grewia (*Grewia* spp.), and Commiphora (*Commiphora* spp.) trees interspersed with open grassy areas. The area is hilly and ranges in elevation between 800–1400 meters. Annual rainfall averages ~500 mm [59] with most rainfall concentrated in the months of December–April. There are several year-round springs distributed across the Kideru Ridge, which form important water sources for people, livestock, and wildlife during the dry season. We do not share exact geographic coordinates of the study location so as to minimize the chance for misuse of these data [60].

### Camera trap survey

**Fig 1:**
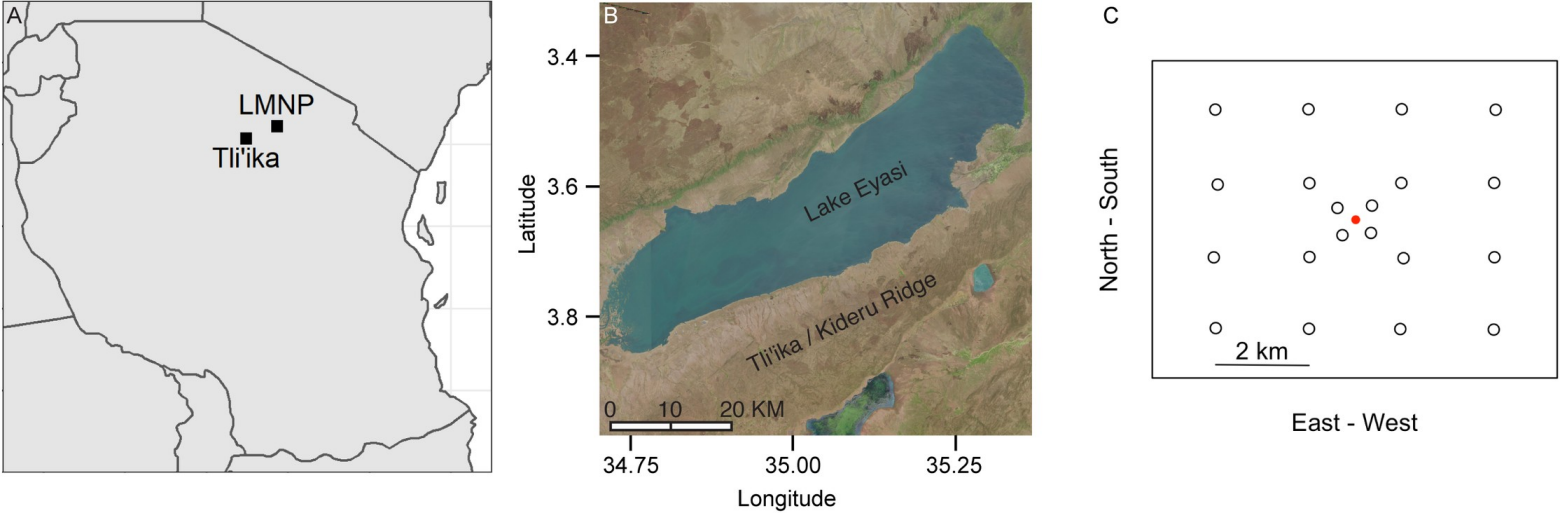
Map of the study area. A) Tanzania, and the location of Tli’ika and Lake Manyara National Park (LMNP); B) The region of Tli’ika in the Lake Eyasi area of northern Tanzania; C) The spatial distribution of 20 installed camera traps, centered upon the Hadza camp indicated in red.

One of the long-term goals of this project is to examine relationships between Hadza landscape use and patterns of wildlife distribution, and so we designed the grid to provide a random sample of locations that was stratified across different rates of occupation and use during people’s normal patterns of central-place foraging. GPS data collected between 2006 and 2018 [61] provided us with an empirical model of Hadza movement patterns, and its variation as a function of radial distance from camp. Therefore, we designed a master grid that was composed of 16 cameras spaced 2 km apart, and a sub-grid closer to the Hadza camp, composed of 4 cameras, spaced 1 km apart, as shown in Fig 1C. To implement this grid design, we traveled across the survey area with three Hadza research assistants and a District Government wildlife officer, and deployed the cameras onto suitably robust, mid to large-sized trees at a height of about 60 cm. A total of 20 camera traps (Bushnell Trophy Cam, infrared flash) were deployed and they operated for a maximum of 141 days, from March to July of 2018. Most camera traps remained operational at each site for the entire study period. However, five of the cameras were stolen, and then replaced with new cameras placed in close proximity to the original locations. At one site, cameras were repeatedly stolen and we did not obtain any data from this location. Camera traps were protected by custom-made, locked metal cases and attached to trees using screws. We set the cameras so that they would record a maximum of one picture per minute. This set-up ensures relatively long battery life, and reduces data storage requirements without compromising detection probability [62]. Photos were entered and stored in the open source software program Camelot [63]. For each picture containing an animal, we identified the species using a field guide [64], counted the number of individuals per frame. Because it was impractical to identify individual animals and thus difficult to define a single contact in the camera detection zone, we removed pictures of the same species that were taken within one hour of the initial picture of the same species prior to analysis to avoid pseudo-replications at each location [65,66].

### Data analyses

We estimated species richness for all mammals with a mass equal to or larger than the northern lesser galago (*Galago senegalensis*; 0.23 kg). To assess whether sampling effort was sufficient, we fit species accumulation curves using the software EstimateS 9.1 [67] and visually assessed if the species richness–sampling effort relationship reached a plateau. We calculated bootstrapped 95% confidence intervals using 100 resamples. We estimated landscape-level species richness as a function of the number of camera trap stations utilized, and estimated site-specific species richness as a function of the number of camera trap nights.

To assess the relative abundance of mammal species we computed a relative abundance index (*RAI*) which scales the number of independent camera events to 100 trap nights; in line with other camera trap research, we omitted consecutive captures of the same species that were within one hour of the initial capture of the same species unless separated by camera trap events of other species [66,68,69]. Please note that this approach scores the presence or absence of a species, and does not account or adjust for differences in detectability, or weight scores by the number of individuals seen in a frame. We calculated RAI for each species and for each camera trap station, and we computed aggregated RAI measures for herbivores, wildlife, and livestock for each camera trap station. We report summary statistics of these RAI scores across all camera trap stations including their means, medians, and 95% confidence intervals. We compared the relative abundance index (RAI) of mammal species in Tli’ika to RAI estimates derived from a similar camera trap study conducted from June 2016 to June 2017 (a total of 6,479 camera trap nights) in nearby Lake Manyara National Park (LMNP) [65]. LMNP is less than 100 km from our monitoring area and strong restrictions on hunting and livestock keeping are in place [70]. We consider the role of these anthropogenic influences and the environmental differences between our survey area and LMNP in the discussion.

In the LMNP survey, we used 46 camera trap locations, spaced out in a systematic 1.5 km grid and used LTL Acorn 5210A cameras (Zhuhai Ltl Acorn Electronics Co Ltd., Guangdong, China). To ensure that the two datasets were comparable, we used the same camera trap settings in Tli’ika (i.e. setting camera speed to a maximum of one picture per minute, medium sensitivity) and applied the same one hour threshold for defining independent camera events [65]. To compare the rate of camera trap detections between the two areas, we fit negative binomial regression models using the MASS package [71]. A separate model was fit to the data for each species. In these models, the number of detections at each camera trap location was the dependent variable, while study location (LMNP vs. Tli’ika) was the predictor variable, and the log number of camera trap nights was an offset variable representing the sampling duration at each camera trap location. For each species comparison, we report *p*-values derived from a likelihood ratio test comparing an intercept-only null model to a model as described above, which include a term for study location. The relevant data for this study (i.e. species specific capture events at the resolution of each camera trap station) are deposited in the supplementary material (S1 Data).

The 19 functioning camera traps operated for a range of 68–140 camera trap nights (69 to 141 camera trap days) from March 1 to July 21, 2018 with an average of 115 nights (116 days) per site. Cumulatively, a total of 2,182 camera trap nights (2,206 trap days) were collected. We identified at least 36 wild mammal species (Table 1). Image quality did not allow for a reliable distinction between Cape hares (*Lepus capensis*) and scrub hares (*Lepus saxatilis*) and we hence combined these events as hares. The cameras also recorded domesticated animals including dogs (*Canis familiaris*), cattle (*Bos taurus*), donkeys (*Equus asinus*), sheep (*Ovis aries*) and goats (*Capra hircus*). For estimating relative livestock densities, we combined sheep and goat into one category.

**Table 1:** Mammal species (listed in descending order of relative abundance) detected during a camera trap survey conducted in Tli’ika, Lake Eyasi region, northern Tanzania in 2018.

Species	Scientific name	BM (kg)	# of events	RAI	RAI 95% CI	Conservation status
**Wildlife**						
Kirk’s dik-dik	*Madoqua kirkii*	4.5	478	20.34	9.25–31.42	Least Concern
Common genet	*Genetta genetta*	2	106	4.65	1.73–7.56	Least Concern
Greater kudu	*Tragelaphus strepsiceros*	215	102	4.56	1.13–7.99	Least Concern
Impala	*Aepyceros melampus*	52.75	79	4.00	1.75–6.25	Least Concern
Vervet monkey	*Chlorocebus pygerythrus*	4.75	54	2.64	0.32–4.96	Least Concern
Bushbuck	*Tragelaphus scriptus*	35.75	46	2.21	0.67–3.74	Least Concern
Spotted hyena	*Crocuta crocuta*	62.5	42	2.06	0.81–3.31	Least Concern
Black-backed jackal	*Canis mesomelas*	8.25	45	1.99	0.22–3.76	Least Concern
Large-spotted genet	*Genetta tigrina*	2.15	29	1.32	0.20–2.45	Least Concern
Hare	*Lepus spp*.	2.15	30	1.28	0.16–2.40	Least Concern
Bushpig	*Potamochoerus larvatus*	75	28	1.27	0.29–2.26	Least Concern
Bush hyrax	*Heterohyrax brucei*	1.85	32	1.22	0.00–3.61	Least Concern
Northern lesser galago	*Galago senegalensis*	0.23	24	0.91	0.00–1.98	Least Concern
African civet	*Civettictis civetta*	11	23	0.90	0.00–2.30	Least Concern
Bushy-tailed mongoose	*Bdeogale crassicauda*	1.65	21	0.84	0.00–2.25	Least Concern
Bush duiker	*Sylvicapra grimmia*	17.5	16	0.83	0.07–1.60	Least Concern
Maasai giraffe	*Giraffa tippelskirchi*	1017.5	16	0.77	0.00–1.92	Vulnerable
Crested porcupine	*Hystrix cristata*	15	15	0.74	0.03–1.46	Least Concern
White-tailed mongoose	*Ichneumia albicauda*	4	16	0.68	0.00–1.41	Least Concern
Olive baboon	*Papio anubis*	15.25	15	0.61	0.27–0.96	Least Concern
Slender mongoose	*Herpestes sanguineus*	0.48	15	0.60	0.15–1.06	Least Concern
Klipspringer	*Oreotragus oreotragus*	11.78	11	0.52	0.00–1.09	Least Concern
Bat-eared fox	*Otocyon megalotis*	4	11	0.43	0.00–0.87	Least Concern
Warthog	*Phacochoerus africanus*	68.75	8	0.41	0.00–1.13	Least Concern
Striped hyena	*Hyaena hyaena*	30	8	0.37	0.13–0.61	Near Threatened
Honey badger	*Mellivora capensis*	10.38	7	0.32	0.00–0.66	Least Concern
Leopard	*Panthera pardus*	41.5	7	0.32	0.00–0.80	Vulnerable
Caracal	*Caracal caracal*	12.75	8	0.30	0.00–0.63	Least Concern
Aardwolf	*Proteles cristata*	11	7	0.28	005–0.50	Least Concern
Dwarf mongoose	*Helogale parvula*	0.32	4	0.19	0.00–0.40	Least Concern
African wild cat	*Felis silvestris*	4.48	4	0.18	0.00–0.40	Least Concern
African bush elephant	*Loxodonta africana*	4125	4	0.17	0.00–0.35	Vulnerable
Aardvark	*Orycteropus afer*	65	2	0.08	0.00–0.18	Least Concern
Eland	*Taurotragus oryx*	475	1	0.08	0.00–0.22	Least Concern
Plains zebra	*Equus quagga*	241.25	1	0.04	0.00–0.11	Near threatened
African wild dog	*Lycaon pictus*	37.5	1	0.04	0.00–0.11	Endangered
**Livestock**						
Cattle	*Bos indicus*	253	180	9.00	5.06–12.94	
Domestic dog	*Canis lupus familiaris*	14.7	46	1.84	0.00–4.64	
Donkey	*Equus africanus asinus*	137.5	28	1.33	0.10–2.57	
Sheep and goat	*Capra* spp. & *Ovis* spp.	25	5	0.29	0.00–0.62	

For each species, we report the body mass (BM), the number of independent photo events (Events), the mean relative abundance index (RAI: Independent events/100 trap nights) and associated 95% confidence intervals, and the IUCN conservation status.

## Results

### Mammal species richness in Tli’ika

**Fig 2:**
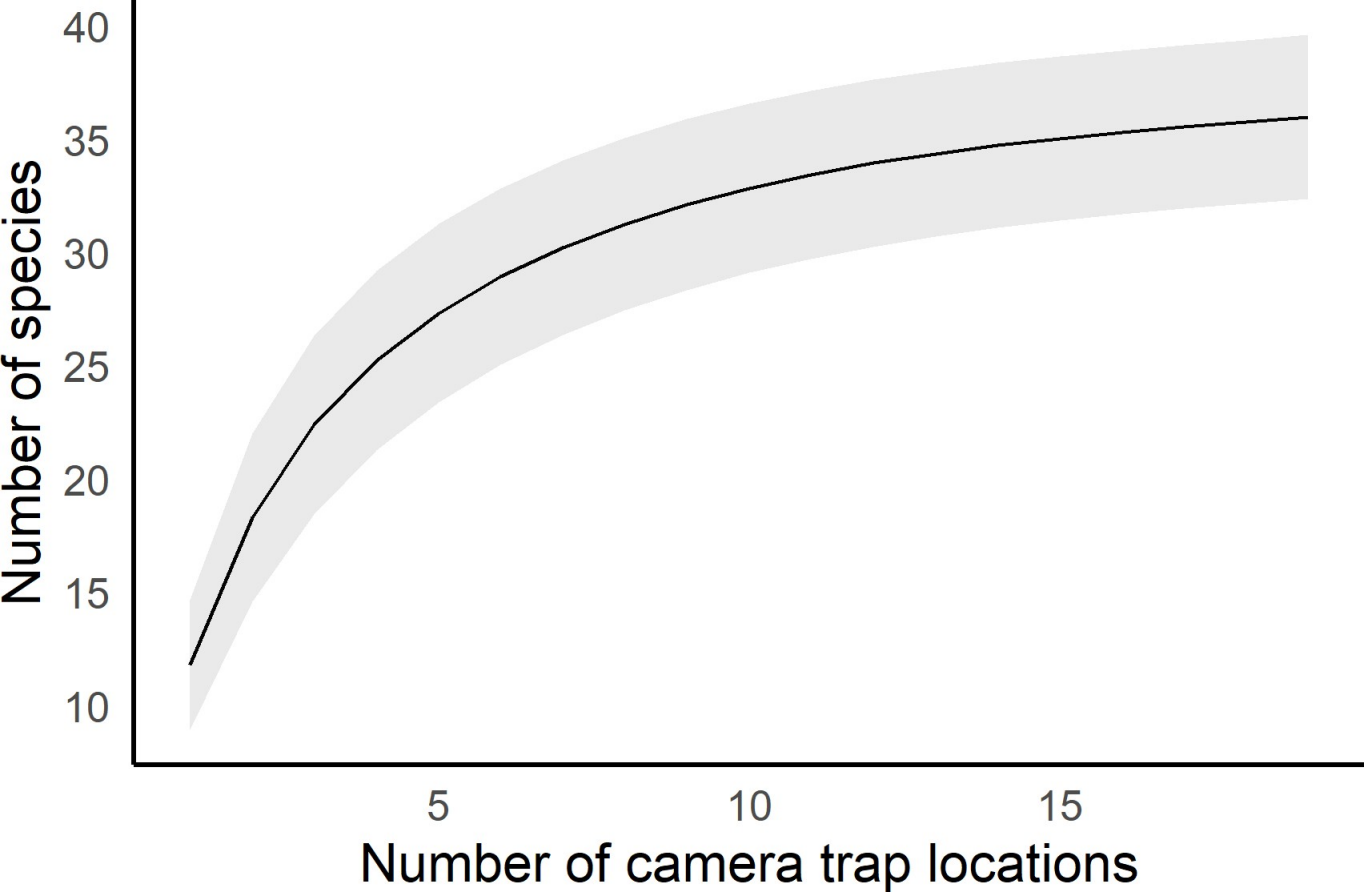
Landscape scale estimate of mammal species richness in Tli’ika, northern Tanzania. Species richness estimates are plotted against camera trap stations; the grey-shaded area indicates 95% confidence intervals of the mean.

Among the 36 mammal species, we detected 12 ungulate species (Kirk’s dik-dik, bush duiker, klipspringer, bushbuck, impala, warthog, bushpig, greater kudu, zebra, eland, giraffe, and elephant), 17 carnivore species (common and large-spotted genets, bushy-tailed mongoose, slender mongoose, white-tailed mongoose, dwarf mongoose, black-backed jackal, African civet, bat-eared fox, honey badger, caracal, aardwolf, African wild cat, spotted and striped hyenas, leopard and African wild dog), three primate species (lesser galago, vervet monkey and olive baboon) as well as bush hyraxes, hares, porcupines and aardvarks (for scientific names, see Table 1). The accumulation curve of the landscape-level mammal species richness was nearly asymptotic, suggesting that sampling was near complete (Fig 2). The average number of mammal species detected per camera site was 11.5, ranging from three to 20 species (S1 Fig). The species accumulation curves of six of the 19 sites (sites # 1, 3, 5, 7, 18, 20) appear to have approached an asymptote by the end of the operational period, while accumulation curves of the remaining 13 sites show an increasing trend even by the end of the operational period (S1 Fig).

### Relative abundance of mammal species in Tli’ika

Relative abundance indices (RAI) of wildlife species varied by c. three orders of magnitude, from a mean of 0.04 capture events per 100 trap-nights (African wild dog) to 20.34 capture events (Kirk’s dik-dik) (Table 1). Among all wild species, next to Kirk’s dik-diks, greater kudu, impala, vervet monkey and bushbucks were relatively abundant in the study area. Among the carnivores, the common genet reached highest relative abundance, followed by spotted hyena, black-backed jackal, and large-spotted genet (Table 1). The relative abundance index of cattle was 9.0, the second highest of any species. Among livestock species, cattle were captured most frequently, followed (by a large margin) by domestic dogs, donkey, and sheep and goats.

### Relative wildlife abundances in Tli’ika and Lake Manyara NP

**Fig 3:**
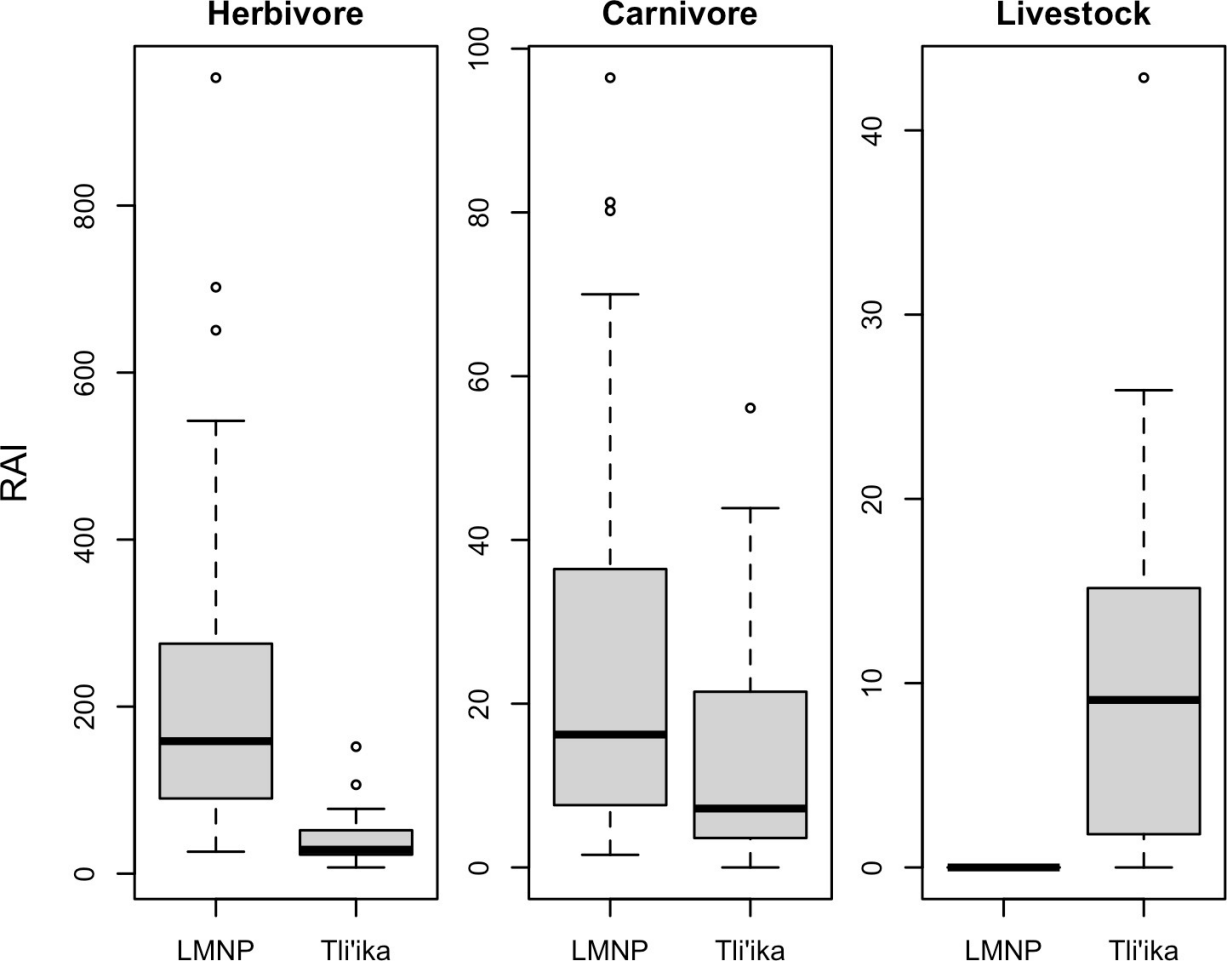
Boxplots, comparing relative abundance indices (RAI; camera trap events/100 trap nights) across functional groups of mammals in Tli’ika and Lake Manyara National Park (LMNP), in northern Tanzania.

To compare gross levels of animal detections at the two sites, we first summed the species-specific RAI indices at each camera trap station across all wild herbivores (herbivore RAI), all wild carnivores (carnivore RAI), and all livestock (livestock RAI), and then calculated the median values for each RAI metric across the camera trap stations of the two sites (Fig 3). The median wild herbivore RAI in Tli’ika was 28.7 detections per 100 camera trap nights, while in LMNP, the median RAI was 452% higher, at 158.6. The median wild carnivore RAI in Tli’ika was 7.2, and that of LMNP 16.2, i.e. 126% higher. These data indicate that both wild carnivores and herbivores were more abundant in LMNP (Fig 3). In contrast, camera traps in Tli’ika recorded a substantial frequency of livestock species (median livestock RAI = 9.1), while in LMNP, camera traps did not detect any livestock.

**Fig 4:**
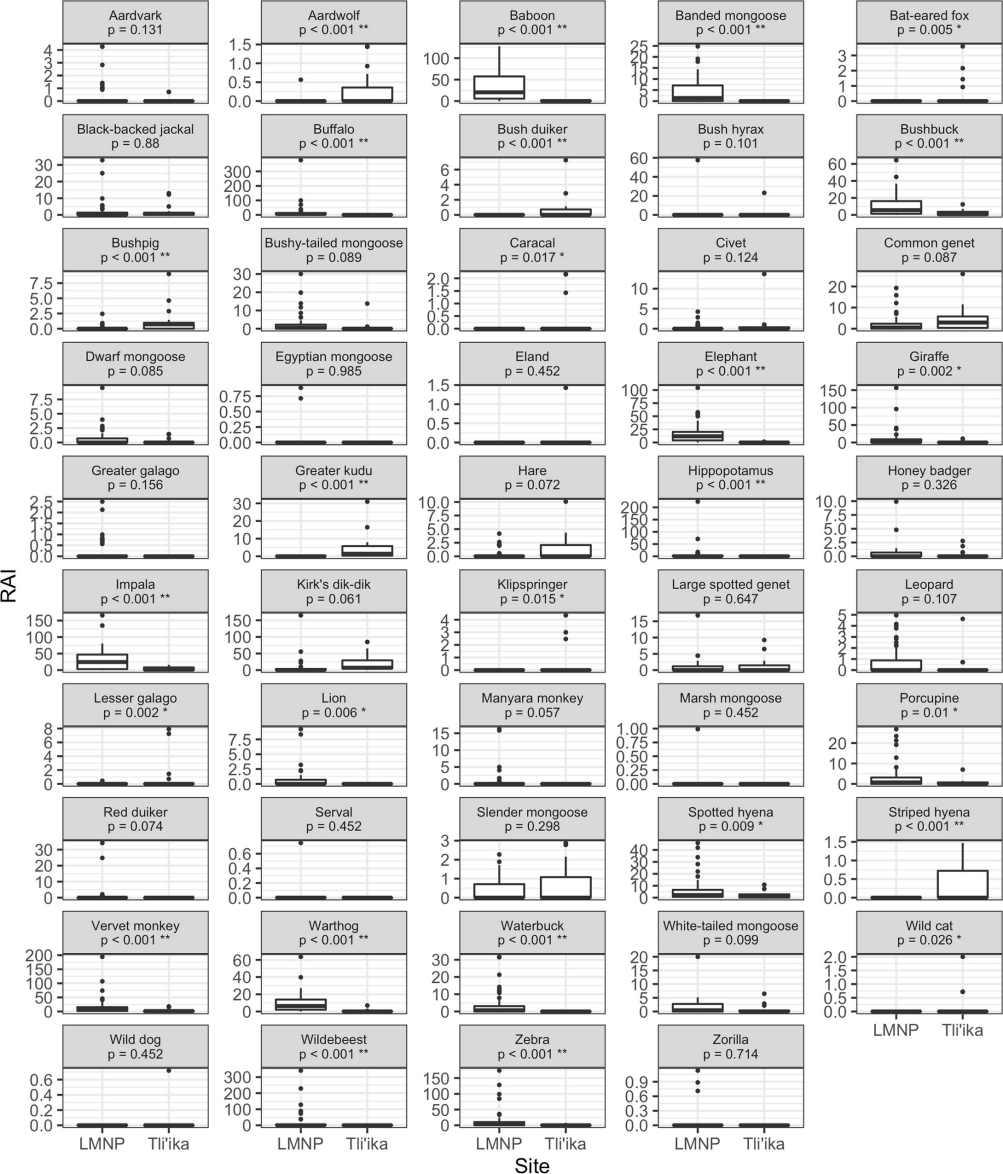
Boxplots, comparing relative abundance indices (RAI; camera trap events/100 trap nights) for mammal species in Tli’ika and Lake Manyara National Park (LMNP), in northern Tanzania. The associated p-value is derived from a likelihood-ratio test which compared a model with a site effect (Tli’ika vs. LMNP) to an intercept-only model (S2 Data).

Negative binomial regression modeling indicates that the following species had significantly higher RAI (or occurred exclusively) in LMNP compared to Tli’ika (Fig 4; S2 Data): olive baboon, banded mongoose, buffalo, elephant, giraffe, hippopotamus, impala, lion, Manyara monkey (*Cercopithecus mitis manyaraensis*), porcupine, red duiker (*Cephalophus natalensis*), spotted hyena, vervet monkey, warthog, waterbuck, wildebeest (*Connochaetes taurinus*) and zebra. On the other hand, aardwolf, bat-eared fox, bush duiker, bushpig, caracal, greater kudu, klipspringer, lesser galago, striped hyena, and wild cat had greater relative abundances in Tli’ika compared to LMNP or occurred exclusively in Tli’ika. All of these later species, with the exception of striped hyena and aardwolf, are readily pursued by Hadza hunters when encountered, but prey offtake rates vary considerably. Remaining RAI values did not differ significantly between the two sites (Fig 4; S2 Data). It is important to note that zebra and eland, which are historically and culturally important prey items for Hadza hunters [72,73], were detected at the lowest rates of all species.

## Discussion

This camera trap study documents high mammal species richness, relatively low wild mammal detection rates, and high livestock detection rates in an area used by Hadza hunter-gatherers and Datoga pastoralists. Both wild herbivores and carnivores were detected at lower rates in Tli’ika than in nearby Lake Manyara National Park, suggesting that wildlife densities are generally lower in the Hadza-occupied region. This study also provides important data for understanding the historical and ecological contexts of contemporary Hadza hunting practices and diet.

### Wildlife persistence in a human-dominated landscape

Research on the patterns and chronology of Anthropogenic defaunation suggest that this process can be classified in three phases: 1. wildlife exploitation using simple traditional technologies; 2. adoption of more sophisticated technologies to exploit wildlife; 3. habitat conversion to urban or agricultural space [21,74]. As these anthropogenic factors intensify, particularly large-bodied mammal species (which often require large home ranges) are often the first species to become locally extinct [10,11,26,75]. Relatively few species are known to have been extirpated from Tli’ika in recent history; black rhinoceros (*Diceros bicornis*) were extirpated in the 1970’s [58], and while buffalo were known to occur within our study location in the 1980’s, they were not detected in our camera trap survey. As recently as 2007, BW had observed ground pangolin (*Smutsia temminkii*) in the survey area, but has not seen one since, despite yearly visits and extensive travels throughout the area. Trade in pangolins has occurred in this region, and perhaps caused this species’ local extirpation.

Multiple factors likely help to sustain a generally species-rich mammal community, including predominant use of traditional weapons to hunt wildlife, low human population density, a large extent of relatively intact habitat (the Kideru ridge provides c. 800 km^2^ of habitat), and sustained connectivity to the Ngorongoro Conservation Area [32,76]. While subsistence hunting likely increases the risk of local extinctions, the survey area is notable for being quite remote from roads or agricultural villages, and currently having low human population density overall. Thus, the current magnitude of local anthropogenic drivers of defaunation is likely not as extreme as in other areas in Tanzania. It is important to note that the Hadza community in the area has helped develop local land-use by-laws to limit the spatial extent of agricultural development and land conversion. In addition, small groups of village game scouts carry out periodic anti-poaching patrols, limiting the pressure of hunters using snares and firearms that would otherwise be manifest by non-Hadza. Anti-poaching efforts and direct payments to villages in return for effective habitat conservation are financed by revenue gained from REDD+ mechanisms, facilitated and organized by a Tanzanian NGO, Carbon Tanzania [34].

Although camera traps recorded a remarkable number of mammal species, we did not capture some of the prominent species that are known to occur either in the Yaeda Valley or other portions of the Kideru Hills, which have been detected in previous walking surveys of those areas: e.g. African lions (*Panthera leo*), cheetah (*Acinonyx jubatus*), wildebeest (*Connochaetes taurinus*), Thomson’s gazelle (*Eudorcas thomsonii*) and buffalo (*Syncerus caffer*) [32]. Some of these non-detections are likely due to mismatches between the camera trap grid and the habitat selection of the species in the study area (e.g. wildebeest and Thomson’s gazelle which mainly occur in the grasslands of the Yaeda valley). Lions are frequently heard in Tli’ika but their populations densities are very low, and so while they are present, were not detected by the camera traps [65]. On a positive note, our photo capture of the wild dog (*Lycaon pictus*) confirms the presence of this endangered species in the study area [32,64]. These comparisons underline the general consensus that a combination of multiple survey methods is necessary to capture an entire mammal community in a given area [77]. An added benefit of using camera traps is the ability to identify animals to the species level in almost all cases. Based on indirect signs, this may be difficult to achieve even for highly skilled trackers [78]. For example, our data allowed identification of mongoose to the species-level (not possible in [31]) and this extended the known range of the bushy-tailed mongoose (*Bdeogale crassicauda*) [64].

In contrast to high levels of observed species richness, relative abundance of several wildlife species were markedly lower in Tli’ika than in the fully protected LMNP (Figs 3 and 4). Wildlife densities are primarily determined by bottom-up (i.e. resource availability) processes but top-down processes (e.g. predation, disease) can lower their resource-determined densities as well [79,80]. While our observational study only provides a snapshot in time and thus prevents elucidating the processes leading to the observed species densities, we discuss some underlying hypotheses.

Importantly, net primary productivity (NPP) in the reference study area (LMNP) was greater than in Tli’ika. Computed using MODIS satellite imagery (Terra Net Primary Production Yearly Global 1km (MODIS/055/MOD17A3), between the years 2001 and 2015, NPP averaged 9,909 kg carbon/m^2^ (SD = 1126) in LMNP, while in the Tli’ika camera-trapping zone, it averaged 6,396 kg carbon/m^2^ (SD = 704). The two locations receive similar amounts of precipitation (~500 mm/year) but the amount of runoff and groundwater available in LMNP is greater because it draws from a larger and higher elevation watershed [81]. Given the positive relationship between NPP and ungulate densities in East Africa—this difference may explain some of the observed contrasts in relative abundances between Tli’ika and LMNP [82], but hunting pressures and competition with Datoga livestock in Tli’ika are likely to be stronger factors contributing to the large observed differences.

For several species, it is unlikely that hunting by Hadza caused the observed low abundances. In general, Hadza hunters fear elephants (*Loxodonta africana*) and are very wary of African buffalo (*Syncerus caffer*); the former are not hunted whatsoever, the later, only occasionally [72]. In addition, Hadza hunters do not consider hyena species, aardwolf, civets, or jackals to be suitable for human consumption. Civets and jackals are rarely hunted, but hyenas are considered a potentially dangerous animal and are occasionally killed [40]. Interestingly, against a trend of generally lower mammal detections in Tli’ika, black-backed jackals and leopards were found at similar relative abundances in Tli’ika and LMNP. The detection rate of caracals was also relatively high. Likely, the persistence of these carnivore species is facilitated by their wide feeding niches and the relative high abundance of small prey species such as hyraxes and dik-diks [83–85].

Lower relative abundances of many mammal species in Tli’ika does not mean that Hadza hunting practices are solely responsible. Eight out of the 10 species with detection rates significantly *higher* in Tli’ika than in LMNP are prey that the Hadza actively pursue when encountered [40,59], which suggests that Hadza prey choice patterns cannot be the sole determinant of detection rate differences between the sites. Top down regulation of herbivore populations may differentially affect species of varying body masses [86] and its strength may be conditional on primary productivity [87]. Long-term monitoring of the mammal community is needed to elucidate Hadza hunting effects on mammal populations.

Resource competition between livestock and wildlife is a local concern, and may also contribute to lower wildlife densities. Zebra and eland were detected at the lowest rates of all species in Tli’ika. These two species are both preferred prey for Hadza hunters and while eland are flexible in their diet [88], zebra are obligate grazers [89] that require large quantities of grass [90]. These species are likely in direct competition with livestock. Livestock reduce grass availability, and particularly transform the areas surrounding water sources through trampling and intense grazing. This competition occurs in the dry season, when the low lying areas surrounding Tli’ika are often completely grass-free, and even less suited for zebra and eland. Though we cannot disentangle the influences of these multiple factors in this study, it is surely notable that densities of livestock species (particularly cattle) in this area were high. For example, walking transects carried out from 2015 to 2018 in the lower portions of the Kideru Hills, directly adjacent to our camera trap grid suggest that mean cattle and goat/sheep densities ranged from 8–51 and 0–4 ind. km^-2^, respectively [32]. High livestock densities and their projected impacts on wildlife communities have implications for local land use planning, and should be a target for continued ecological monitoring. Ideally, experimental or quasi-experimental studies such as livestock exclusion experiments or studies on livestock-wildlife interactions can shed more light on the relative contribution of each of these factors on wildlife population trajectories [91].

Our survey demonstrates the possibility for coexistence of wildlife and subsistence hunters in shared landscapes, but the generally low detection rates of wildlife in Tli’ika are a concern for conservation and sustainable hunting. Additional stressors on wildlife populations that could be caused by agricultural expansion, new forms of infrastructure or land use (e.g., road construction, mineral or petrochemical exploitation), likely entail high risks to the low-density wildlife populations currently residing in the area which provide major ecosystem services to the Hadza community.

### Wildlife monitoring in human dominated areas

The importance of assessing wildlife communities in human-dominated areas of sub Saharan Africa is increasingly being recognized [28,29,42,92]. However, wildlife populations are often assessed using methods that rely on direct sightings by observers in the framework of strip or line distance sampling surveys [27,93]. While these estimates may be reliable for diurnal and abundant species under some circumstances, these methods fail to detect many species [65] and may underestimate densities of hunted species [94]. As case in point, the frequency of direct encounters during walking transects in an adjoining study area [32], provided very few direct encounters of most wildlife species. Rates of camera trap detections are frequently employed as proxy for wildlife densities, but there are biases and uncertainties to be aware of. Bias could arise because this method does not explicitly account for differences in detectability [95], which could arise, for example, due to site- and species-specific differences in ranging patterns and camera viewsheds. Nevertheless, camera trapping has provided a scalable means to assess wildlife communities in human-exploited areas, and the development of new analytical methods [96], and increasingly extensive sampling efforts [97] are likely to continue.

A major, largely unresolved challenge for camera trap surveys in human-dominated areas are vandalism and theft, which constitutes a major data and financial loss [98]. Nevertheless, we hope that continuous community engagement will facilitate longitudinal mammal monitoring in the area, and provide data valuable to local land use planning as well as fundamental research in anthropology and ecology.

### Contemporary Hadza hunting, diet, and reconstructions of the past

As this is the first direct assessment of the medium- and large-sized mammal community in an area regularly hunted by the Hadza, it will inform future anthropological research investigating the Hadza’s foraging economy, including patterns of prey choice, landscape utilization, and the sustainability of prey off-take. The high RAI of livestock in the camera trap grid and high livestock densities across the wider area [32] raises several important issues. The presence of domestic livestock and the generally low detection rates of wildlife call into question the assumption that contemporary Hadza hunting practices should be considered representative of foraging economies that would have prevailed prior to the neolithic revolution [99]. High livestock densities are associated with decreases in wildlife biomass across Africa [43]. Prior claims that the Yaeda-Tli’ika region sustains densities of wild mammals similar to that of national parks with similar rainfall levels [51] are not consistent with our data. The question of whether contemporary hunter-gatherers occupy marginal habitats has been addressed in recent studies by comparing the net primary productivity (NPP) of forager-occupied habitats and farmer-occupied habitats [54,55]. Our study leads us to wonder whether these studies have adequately assessed habitat quality from the perspective of hunter-gatherers because the abundance of animal prey is not directly estimated in NPP-based studies. NPP is important for explaining many ecological differences, and we do not suggest abandoning NPP as a relevant measure in ecological anthropology. But it has its limitations and might not provide a resolution to the "marginal habitat" debate in hunter-gatherer studies. Studies focusing more on historical changes in animal biomass in particular would be very useful for understanding how recent ecological influences have impacted the behavior and social organization of hunter-gatherers.

Several studies of foraging populations (including the Hadza) document food preferences that favor meat consumption relative to plant foods [100–102]. Cross-cultural measures of the return rates (kcal/hr) of a wide variety of different wild foods show that hunted animals have higher average return rates than does the pursuit of key plant foods, including tubers and seeds [103]. These findings suggest that in general, when wildlife is more abundant, more meat will in turn be hunted and eaten. Given the high prevalence of livestock and low abundance of wildlife in the Hadza’s contemporary habitat, it would be reasonable to assume that meat would have comprised a larger fraction of the diet of hunters living in this area in the past, before the movement of pastoralist and agricultural populations into the region. Prey abundance and hunting productivity seem to have declined precipitously in recent times, in the living memory of contemporary Hadza. Blurton-Jones [53] reports that buffalo seemed to “disappear” after 1986 in the region and that “Hadza in Tli’ika attributed the decline in large animals to Datoga fencing the water holes, making the area unattractive to wildlife” (ibid:31). In the absence of robust and comparable wildlife monitoring data, these anecdotes are likely to be the best available evidence for documenting wildlife declines in this region and elsewhere. Although recent increases in some wildlife populations [32] are a positive sign, current wildlife densities are likely well below their historic levels, and research carried out sporadically over short time periods is likely to miss crucial changes in wildlife populations [104]. We think our comparison of wildlife detection rates in Tli’ika and LMNP provides a useful point of reference for contextualizing the possible impact of subsistence hunting and livestock keeping on wildlife populations, but it should be noted that national parks have also experienced precipitious wildlife declines over the last 60 years [5]; thus, even if "park-like" animal abundances were found in the Hadza area, these would likely still be lower than wildlife populations found in the area in the deeper past. Despite the apparently low abundance of wildlife in the Tli’ika area, the Hadza living there continue to hunt and gather, and are ever-resourceful at making a living in their land, under increasingly challenging conditions. It is important to document these recent environmental changes in Hadzaland in order to contextualize their contemporary economic practices, adjust for contemporary biases in reconstructions of the past, and to provide data and training to local community members working to ensure the future viability of subsistence foraging.

## Supporting information

S1 Fig Camera site-specific estimates of mammal species richness in Tli’ika, northern Tanzania. Species richness estimates are plotted against sampling effort; dashed lines indicate 95% confidence intervals of the mean.
(DOCX)

S1 Data Number of independent species-specific detections at camera stations deployed in Lake Manyara National Park (LMNP) and the Kideru ridge (Tli’ika) of northern Tanzania. For each camera location detections of the same species within 60 min of the initial capture were discarded to ensure independence of camera events. “Nights” denotes the number of operational camera trap nights at each camera trap location.
(DOCX)

S2 Data Mean of relative abundance indices (RAI; independent camera trap events/100 camera trap nights) of mammal species in Lake Manyara National Park (LMNP) and the Kideru ridge (Tli’ika) of northern Tanzania. The associated p-value is based on a likelihood ratio test, comparing a model with site effect (LMNP vs. Tli’ika) with an intercept only model.
(DOCX)
