# Supplementary material for: Hunter-Gatherers in context: Mammal community composition in a northern Tanzania landscape used by Hadza foragers and Datoga pastoralists
Source: PLoS One. 2021 May 14;16(5):e0251076. doi: 10.1371/journal.pone.0251076 (PMC8121365; doi:10.1371/journal.pone.0251076)

**S 1 Fig. Camera site-specific estimates of mammal species richness in Tli’ika, northern Tanzania.** Species richness estimates are plotted against sampling effort; dashed lines indicate 95% confidence intervals of the mean.


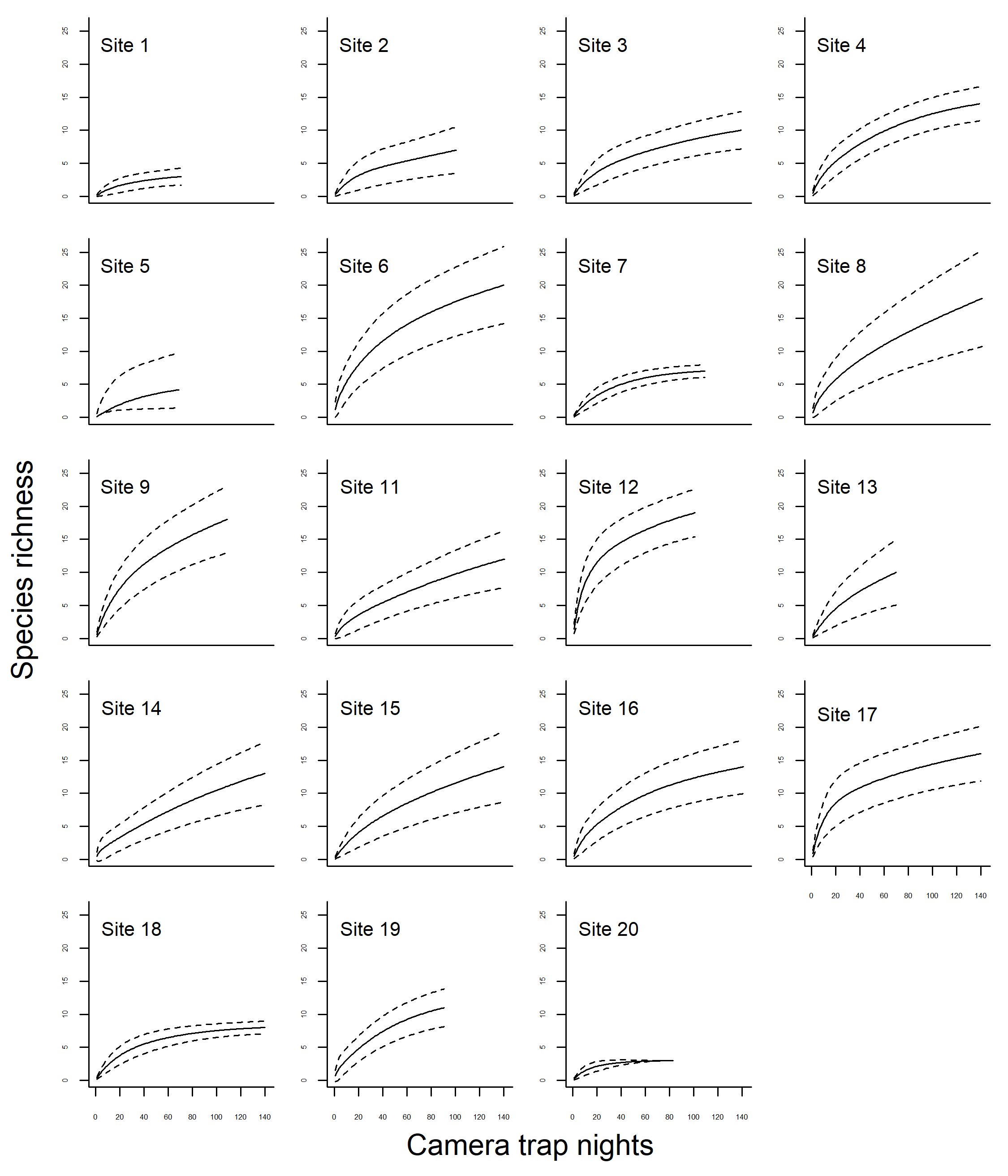

Supplement: S1 Fig — Species richness estimates are plotted against sampling effort; dashed lines indicate 95% confidence intervals of the mean. (DOCX) [file pone.0251076.s001.docx]
