# Supplementary material for: Hunter-Gatherers in context: Mammal community composition in a northern Tanzania landscape used by Hadza foragers and Datoga pastoralists
Source: PLoS One. 2021 May 14;16(5):e0251076. doi: 10.1371/journal.pone.0251076 (PMC8121365; doi:10.1371/journal.pone.0251076)
Supplement: S1 Data — For each camera location detections of the same species within 60 min of the initial capture were discarded to ensure independence of camera events. “Nights” denotes the number of operational camera trap nights at each camera trap location. (DOCX) [file pone.0251076.s002.docx]

**S1 Data. Number of independent species-specific detections at camera stations deployed in Lake Manyara National Park (LMNP) and the Kideru ridge (Tli’ika) of northern Tanzania.** For each camera location detections of the same species within 60 min of the initial capture were discarded to ensure independence of camera events. “Nights” denotes the number of operational camera trap nights at each camera trap location.

| Station | Nights | Site | Aardvark | Aardwolf | Civet | Elephant | Lion | Banded mongoose | Black-backed jackal | Manyara monkey | Bushbuck | Bush hyrax | Bushpig | Bushy-tailed mongoose | Buffalo |
| --- | --- | --- | --- | --- | --- | --- | --- | --- | --- | --- | --- | --- | --- | --- | --- |
| 0 | 141 | LMNP | 6 | 0 | 0 | 74 | 0 | 0 | 0 | 0 | 30 | 0 | 0 | 6 | 26 |
| 1 | 177 | LMNP | 0 | 1 | 0 | 19 | 1 | 0 | 0 | 0 | 1 | 0 | 0 | 1 | 124 |
| 2 | 156 | LMNP | 2 | 0 | 1 | 78 | 5 | 0 | 0 | 0 | 6 | 0 | 0 | 0 | 24 |
| 3 | 188 | LMNP | 0 | 0 | 0 | 32 | 0 | 0 | 0 | 0 | 4 | 0 | 0 | 0 | 15 |
| 4 | 41 | LMNP | 0 | 0 | 0 | 23 | 0 | 0 | 0 | 0 | 3 | 0 | 1 | 0 | 1 |
| 5 | 226 | LMNP | 0 | 0 | 0 | 29 | 1 | 3 | 0 | 1 | 0 | 0 | 0 | 2 | 17 |
| 6 | 208 | LMNP | 0 | 0 | 0 | 24 | 0 | 7 | 0 | 0 | 5 | 0 | 0 | 1 | 12 |
| 7 | 195 | LMNP | 2 | 0 | 0 | 27 | 0 | 0 | 0 | 0 | 1 | 0 | 0 | 1 | 9 |
| 8 | 141 | LMNP | 0 | 0 | 4 | 15 | 0 | 1 | 0 | 0 | 37 | 0 | 1 | 0 | 5 |
| 9 | 226 | LMNP | 0 | 0 | 0 | 2 | 0 | 56 | 1 | 0 | 1 | 0 | 0 | 8 | 852 |
| 10 | 141 | LMNP | 0 | 0 | 6 | 147 | 13 | 27 | 5 | 0 | 2 | 0 | 0 | 0 | 55 |
| 12 | 135 | LMNP | 0 | 0 | 2 | 56 | 3 | 16 | 1 | 0 | 34 | 0 | 0 | 3 | 16 |
| 15 | 140 | LMNP | 0 | 0 | 1 | 24 | 1 | 18 | 5 | 0 | 10 | 0 | 0 | 12 | 46 |
| 16 | 141 | LMNP | 2 | 0 | 0 | 14 | 2 | 8 | 8 | 0 | 0 | 0 | 0 | 2 | 18 |
| 18 | 141 | LMNP | 0 | 0 | 0 | 0 | 0 | 1 | 0 | 0 | 9 | 0 | 0 | 0 | 0 |
| 20 | 175 | LMNP | 0 | 0 | 0 | 14 | 4 | 2 | 0 | 0 | 9 | 0 | 0 | 11 | 6 |
| 21 | 143 | LMNP | 0 | 0 | 0 | 4 | 0 | 1 | 0 | 0 | 0 | 0 | 0 | 0 | 7 |
| 24 | 118 | LMNP | 0 | 0 | 0 | 17 | 0 | 2 | 0 | 2 | 76 | 0 | 0 | 0 | 0 |
| 25 | 225 | LMNP | 0 | 0 | 0 | 4 | 0 | 0 | 0 | 2 | 53 | 0 | 1 | 0 | 1 |
| 26 | 141 | LMNP | 0 | 0 | 0 | 35 | 0 | 2 | 0 | 0 | 45 | 0 | 0 | 0 | 7 |
| 30 | 158 | LMNP | 0 | 0 | 0 | 2 | 1 | 11 | 0 | 0 | 1 | 0 | 0 | 0 | 9 |
| 31 | 141 | LMNP | 0 | 0 | 0 | 8 | 0 | 0 | 0 | 0 | 10 | 0 | 0 | 1 | 1 |
| 32 | 85 | LMNP | 0 | 0 | 0 | 20 | 0 | 5 | 2 | 0 | 38 | 0 | 0 | 0 | 14 |
| 36 | 80 | LMNP | 0 | 0 | 0 | 17 | 0 | 0 | 0 | 0 | 11 | 0 | 0 | 24 | 4 |
| 37 | 190 | LMNP | 0 | 0 | 0 | 11 | 2 | 18 | 5 | 0 | 17 | 0 | 0 | 1 | 6 |
| 38 | 71 | LMNP | 0 | 0 | 0 | 6 | 0 | 6 | 7 | 0 | 0 | 0 | 0 | 0 | 1 |
| 40 | 101 | LMNP | 0 | 0 | 1 | 30 | 0 | 7 | 0 | 0 | 9 | 0 | 0 | 20 | 11 |
| 43 | 306 | LMNP | 0 | 0 | 0 | 23 | 0 | 8 | 0 | 0 | 18 | 0 | 0 | 4 | 3 |
| 46 | 125 | LMNP | 0 | 0 | 0 | 72 | 1 | 18 | 0 | 2 | 24 | 0 | 0 | 0 | 10 |
| 47 | 160 | LMNP | 0 | 0 | 0 | 3 | 0 | 9 | 5 | 0 | 2 | 0 | 0 | 1 | 159 |
| 50 | 226 | LMNP | 0 | 0 | 1 | 9 | 0 | 0 | 0 | 0 | 8 | 130 | 0 | 14 | 8 |
| 51 | 96 | LMNP | 0 | 0 | 0 | 1 | 8 | 12 | 24 | 0 | 0 | 0 | 0 | 0 | 26 |
| 54 | 106 | LMNP | 3 | 0 | 0 | 17 | 0 | 1 | 0 | 0 | 39 | 0 | 0 | 2 | 9 |
| 55 | 140 | LMNP | 0 | 0 | 0 | 6 | 0 | 25 | 2 | 0 | 3 | 0 | 0 | 1 | 28 |
| 59 | 140 | LMNP | 0 | 0 | 0 | 17 | 0 | 1 | 0 | 0 | 2 | 0 | 0 | 3 | 5 |
| 63 | 84 | LMNP | 0 | 0 | 0 | 2 | 0 | 0 | 0 | 0 | 8 | 0 | 0 | 2 | 3 |
| 64 | 140 | LMNP | 0 | 0 | 0 | 14 | 0 | 10 | 6 | 0 | 3 | 0 | 0 | 1 | 1 |
| 70 | 132 | LMNP | 0 | 0 | 0 | 19 | 0 | 0 | 0 | 0 | 4 | 0 | 0 | 6 | 8 |
| 71 | 134 | LMNP | 0 | 0 | 0 | 19 | 2 | 18 | 44 | 0 | 0 | 0 | 0 | 0 | 13 |
| 77 | 92 | LMNP | 1 | 0 | 0 | 22 | 2 | 1 | 1 | 0 | 25 | 0 | 0 | 0 | 7 |
| 78 | 123 | LMNP | 0 | 0 | 0 | 0 | 0 | 1 | 7 | 0 | 5 | 0 | 0 | 0 | 14 |
| 82 | 140 | LMNP | 6 | 0 | 0 | 24 | 1 | 2 | 0 | 7 | 17 | 0 | 0 | 0 | 9 |
| 83 | 24 | LMNP | 0 | 0 | 0 | 4 | 0 | 0 | 0 | 0 | 4 | 0 | 0 | 0 | 2 |
| 87 | 101 | LMNP | 1 | 0 | 1 | 23 | 0 | 11 | 0 | 16 | 15 | 0 | 0 | 14 | 4 |
| 90 | 74 | LMNP | 0 | 0 | 0 | 2 | 0 | 0 | 0 | 3 | 0 | 0 | 0 | 0 | 1 |
| 91 | 111 | LMNP | 1 | 0 | 1 | 3 | 0 | 4 | 0 | 18 | 35 | 0 | 1 | 13 | 0 |
| Site 1 | 70 | Tlika | 0 | 0 | 0 | 0 | 0 | 0 | 0 | 0 | 0 | 0 | 0 | 0 | 0 |
| Site 11 | 140 | Tlika | 0 | 0 | 1 | 1 | 0 | 0 | 1 | 0 | 9 | 0 | 1 | 0 | 0 |
| Site 12 | 100 | Tlika | 0 | 0 | 1 | 1 | 0 | 0 | 13 | 0 | 7 | 0 | 9 | 1 | 0 |
| Site 13 | 69 | Tlika | 0 | 0 | 0 | 0 | 0 | 0 | 0 | 0 | 1 | 0 | 0 | 0 | 0 |
| Site 14 | 139 | Tlika | 1 | 0 | 0 | 0 | 0 | 0 | 1 | 0 | 1 | 0 | 0 | 0 | 0 |
| Site 15 | 139 | Tlika | 0 | 0 | 0 | 0 | 0 | 0 | 0 | 0 | 1 | 0 | 4 | 0 | 0 |
| Site 16 | 140 | Tlika | 0 | 0 | 0 | 2 | 0 | 0 | 1 | 0 | 0 | 0 | 1 | 0 | 0 |
| Site 17 | 139 | Tlika | 0 | 1 | 19 | 0 | 0 | 0 | 17 | 0 | 0 | 0 | 1 | 0 | 0 |
| Site 18 | 139 | Tlika | 0 | 0 | 0 | 0 | 0 | 0 | 0 | 0 | 7 | 0 | 0 | 0 | 0 |
| Site 19 | 89 | Tlika | 0 | 0 | 0 | 0 | 0 | 0 | 2 | 0 | 2 | 0 | 0 | 1 | 0 |
| Site 2 | 99 | Tlika | 0 | 0 | 0 | 0 | 0 | 0 | 0 | 0 | 0 | 0 | 1 | 0 | 0 |
| Site 20 | 81 | Tlika | 0 | 0 | 0 | 0 | 0 | 0 | 0 | 0 | 10 | 0 | 0 | 0 | 0 |
| Site 3 | 138 | Tlika | 0 | 2 | 0 | 0 | 0 | 0 | 0 | 0 | 0 | 0 | 1 | 0 | 0 |
| Site 4 | 138 | Tlika | 0 | 0 | 0 | 0 | 0 | 0 | 0 | 0 | 0 | 32 | 1 | 19 | 0 |
| Site 5 | 68 | Tlika | 0 | 0 | 0 | 0 | 0 | 0 | 1 | 0 | 0 | 0 | 0 | 0 | 0 |
| Site 6 | 139 | Tlika | 0 | 2 | 1 | 0 | 0 | 0 | 7 | 0 | 7 | 0 | 2 | 0 | 0 |
| Site 7 | 108 | Tlika | 0 | 0 | 0 | 0 | 0 | 0 | 0 | 0 | 1 | 0 | 5 | 0 | 0 |
| Site 8 | 139 | Tlika | 1 | 1 | 0 | 0 | 0 | 0 | 1 | 0 | 0 | 0 | 1 | 0 | 0 |
| Site 9 | 108 | Tlika | 0 | 1 | 1 | 0 | 0 | 0 | 1 | 0 | 0 | 0 | 1 | 0 | 0 |

| Station | Nights | Site | Common genet | Hippopotamus | Warthog | Porcupine | Dwarf mongoose | Egyptian mongoose | Greater galago | Hare | Honey badger | Impala | Kirk's dik-dik | Large spotted genet |
| --- | --- | --- | --- | --- | --- | --- | --- | --- | --- | --- | --- | --- | --- | --- |
| 0 | 141 | LMNP | 0 | 0 | 16 | 2 | 0 | 0 | 1 | 0 | 0 | 0 | 0 | 2 |
| 1 | 177 | LMNP | 2 | 0 | 8 | 0 | 0 | 0 | 1 | 0 | 0 | 107 | 0 | 0 |
| 2 | 156 | LMNP | 0 | 2 | 35 | 5 | 0 | 0 | 1 | 0 | 0 | 29 | 0 | 0 |
| 3 | 188 | LMNP | 1 | 5 | 28 | 1 | 0 | 0 | 0 | 0 | 0 | 18 | 0 | 0 |
| 4 | 41 | LMNP | 1 | 3 | 4 | 11 | 1 | 0 | 0 | 0 | 0 | 0 | 0 | 1 |
| 5 | 226 | LMNP | 0 | 6 | 17 | 0 | 1 | 0 | 0 | 0 | 1 | 19 | 5 | 0 |
| 6 | 208 | LMNP | 1 | 7 | 27 | 1 | 0 | 0 | 0 | 0 | 1 | 69 | 0 | 1 |
| 7 | 195 | LMNP | 1 | 26 | 29 | 0 | 0 | 0 | 0 | 0 | 0 | 137 | 0 | 0 |
| 8 | 141 | LMNP | 1 | 11 | 25 | 30 | 0 | 0 | 0 | 0 | 0 | 0 | 0 | 2 |
| 9 | 226 | LMNP | 1 | 40 | 10 | 0 | 0 | 2 | 0 | 1 | 0 | 30 | 0 | 1 |
| 10 | 141 | LMNP | 3 | 100 | 13 | 27 | 0 | 0 | 0 | 0 | 14 | 23 | 0 | 1 |
| 12 | 135 | LMNP | 26 | 302 | 22 | 11 | 0 | 0 | 1 | 0 | 2 | 95 | 2 | 6 |
| 15 | 140 | LMNP | 17 | 4 | 89 | 2 | 0 | 0 | 0 | 3 | 1 | 232 | 3 | 4 |
| 16 | 141 | LMNP | 3 | 0 | 56 | 0 | 1 | 0 | 3 | 3 | 1 | 190 | 0 | 1 |
| 18 | 141 | LMNP | 0 | 0 | 0 | 0 | 1 | 0 | 0 | 0 | 0 | 5 | 2 | 0 |
| 20 | 175 | LMNP | 1 | 4 | 27 | 5 | 0 | 0 | 0 | 1 | 0 | 141 | 18 | 3 |
| 21 | 143 | LMNP | 1 | 0 | 1 | 0 | 0 | 0 | 0 | 0 | 1 | 44 | 0 | 0 |
| 24 | 118 | LMNP | 0 | 0 | 4 | 0 | 0 | 0 | 1 | 0 | 0 | 9 | 4 | 0 |
| 25 | 225 | LMNP | 1 | 0 | 3 | 1 | 0 | 0 | 0 | 0 | 2 | 25 | 0 | 0 |
| 26 | 141 | LMNP | 0 | 1 | 11 | 3 | 0 | 0 | 0 | 0 | 0 | 35 | 4 | 0 |
| 30 | 158 | LMNP | 2 | 0 | 2 | 0 | 4 | 0 | 0 | 0 | 1 | 40 | 0 | 0 |
| 31 | 141 | LMNP | 0 | 0 | 1 | 3 | 3 | 0 | 0 | 0 | 2 | 4 | 0 | 0 |
| 32 | 85 | LMNP | 0 | 5 | 18 | 3 | 0 | 0 | 0 | 0 | 0 | 60 | 4 | 0 |
| 36 | 80 | LMNP | 3 | 0 | 10 | 1 | 1 | 0 | 2 | 0 | 1 | 1 | 132 | 2 |
| 37 | 190 | LMNP | 1 | 2 | 4 | 0 | 0 | 0 | 0 | 5 | 0 | 94 | 14 | 0 |
| 38 | 71 | LMNP | 0 | 0 | 10 | 0 | 0 | 0 | 0 | 0 | 0 | 19 | 1 | 0 |
| 40 | 101 | LMNP | 0 | 0 | 11 | 5 | 0 | 0 | 0 | 0 | 0 | 40 | 28 | 1 |
| 43 | 306 | LMNP | 0 | 2 | 6 | 1 | 3 | 0 | 0 | 6 | 1 | 112 | 11 | 0 |
| 46 | 125 | LMNP | 7 | 0 | 10 | 9 | 2 | 0 | 0 | 0 | 6 | 4 | 2 | 2 |
| 47 | 160 | LMNP | 2 | 0 | 21 | 0 | 0 | 0 | 0 | 0 | 0 | 123 | 0 | 0 |
| 50 | 226 | LMNP | 16 | 0 | 4 | 2 | 9 | 0 | 0 | 0 | 1 | 4 | 3 | 10 |
| 51 | 96 | LMNP | 0 | 10 | 26 | 0 | 0 | 0 | 0 | 4 | 1 | 58 | 7 | 0 |
| 54 | 106 | LMNP | 2 | 0 | 6 | 0 | 0 | 0 | 0 | 0 | 0 | 37 | 59 | 0 |
| 55 | 140 | LMNP | 11 | 0 | 2 | 3 | 4 | 0 | 0 | 0 | 0 | 46 | 2 | 3 |
| 59 | 140 | LMNP | 0 | 0 | 5 | 1 | 0 | 0 | 0 | 0 | 0 | 104 | 4 | 0 |
| 63 | 84 | LMNP | 2 | 0 | 1 | 1 | 8 | 0 | 0 | 0 | 0 | 9 | 19 | 1 |
| 64 | 140 | LMNP | 4 | 0 | 8 | 2 | 2 | 1 | 0 | 0 | 1 | 55 | 8 | 1 |
| 70 | 132 | LMNP | 3 | 0 | 3 | 0 | 1 | 0 | 0 | 0 | 0 | 1 | 8 | 1 |
| 71 | 134 | LMNP | 0 | 14 | 21 | 6 | 0 | 0 | 0 | 3 | 0 | 85 | 1 | 0 |
| 77 | 92 | LMNP | 1 | 0 | 2 | 0 | 0 | 0 | 0 | 0 | 0 | 22 | 0 | 1 |
| 78 | 123 | LMNP | 0 | 2 | 3 | 0 | 0 | 0 | 1 | 0 | 0 | 37 | 0 | 0 |
| 82 | 140 | LMNP | 0 | 3 | 3 | 1 | 1 | 0 | 0 | 0 | 0 | 1 | 0 | 0 |
| 83 | 24 | LMNP | 0 | 1 | 1 | 1 | 0 | 0 | 0 | 0 | 0 | 0 | 0 | 0 |
| 87 | 101 | LMNP | 16 | 0 | 7 | 13 | 0 | 0 | 1 | 0 | 1 | 0 | 0 | 17 |
| 90 | 74 | LMNP | 2 | 0 | 0 | 0 | 0 | 0 | 0 | 0 | 0 | 0 | 0 | 0 |
| 91 | 111 | LMNP | 6 | 0 | 1 | 26 | 0 | 0 | 0 | 0 | 0 | 0 | 0 | 0 |
| Site 1 | 70 | Tlika | 0 | 0 | 0 | 0 | 0 | 0 | 0 | 0 | 0 | 7 | 0 | 0 |
| Site 11 | 140 | Tlika | 0 | 0 | 0 | 0 | 0 | 0 | 0 | 0 | 0 | 0 | 29 | 1 |
| Site 12 | 100 | Tlika | 0 | 0 | 7 | 7 | 0 | 0 | 0 | 2 | 0 | 16 | 38 | 0 |
| Site 13 | 69 | Tlika | 2 | 0 | 0 | 1 | 1 | 0 | 0 | 3 | 0 | 1 | 4 | 0 |
| Site 14 | 139 | Tlika | 1 | 0 | 0 | 1 | 0 | 0 | 0 | 0 | 0 | 0 | 64 | 2 |
| Site 15 | 139 | Tlika | 1 | 0 | 1 | 1 | 0 | 0 | 0 | 3 | 0 | 13 | 7 | 1 |
| Site 16 | 140 | Tlika | 0 | 0 | 0 | 0 | 0 | 0 | 0 | 1 | 0 | 5 | 27 | 0 |
| Site 17 | 139 | Tlika | 16 | 0 | 0 | 1 | 0 | 0 | 0 | 14 | 0 | 0 | 24 | 4 |
| Site 18 | 139 | Tlika | 5 | 0 | 0 | 0 | 0 | 0 | 0 | 0 | 0 | 0 | 12 | 0 |
| Site 19 | 89 | Tlika | 4 | 0 | 0 | 1 | 0 | 0 | 0 | 0 | 0 | 0 | 58 | 0 |
| Site 2 | 99 | Tlika | 5 | 0 | 0 | 0 | 0 | 0 | 0 | 0 | 0 | 9 | 7 | 0 |
| Site 20 | 81 | Tlika | 0 | 0 | 0 | 0 | 0 | 0 | 0 | 0 | 0 | 0 | 0 | 0 |
| Site 3 | 138 | Tlika | 1 | 0 | 0 | 0 | 0 | 0 | 0 | 0 | 1 | 2 | 9 | 0 |
| Site 4 | 138 | Tlika | 1 | 0 | 0 | 0 | 2 | 0 | 0 | 0 | 1 | 0 | 3 | 3 |
| Site 5 | 68 | Tlika | 7 | 0 | 0 | 0 | 0 | 0 | 0 | 0 | 0 | 8 | 3 | 1 |
| Site 6 | 139 | Tlika | 36 | 0 | 0 | 1 | 0 | 0 | 0 | 4 | 0 | 10 | 118 | 0 |
| Site 7 | 108 | Tlika | 4 | 0 | 0 | 0 | 0 | 0 | 0 | 0 | 3 | 0 | 0 | 10 |
| Site 8 | 139 | Tlika | 16 | 0 | 0 | 1 | 1 | 0 | 0 | 3 | 0 | 6 | 68 | 0 |
| Site 9 | 108 | Tlika | 7 | 0 | 0 | 1 | 0 | 0 | 0 | 0 | 2 | 2 | 7 | 7 |

| Station | Nights | Site | Leopard | Lesser galago | Giraffe | Marsh mongoose | Baboon | Red duiker | Serval | Slender mongoose | Spotted hyena | Vervet monkey | Waterbuck | White-tailed mongoose |
| --- | --- | --- | --- | --- | --- | --- | --- | --- | --- | --- | --- | --- | --- | --- |
| 0 | 141 | LMNP | 1 | 0 | 0 | 0 | 20 | 35 | 0 | 1 | 2 | 0 | 17 | 0 |
| 1 | 177 | LMNP | 1 | 0 | 1 | 0 | 155 | 0 | 0 | 0 | 12 | 81 | 14 | 5 |
| 2 | 156 | LMNP | 1 | 0 | 0 | 0 | 68 | 0 | 0 | 0 | 14 | 4 | 49 | 0 |
| 3 | 188 | LMNP | 0 | 0 | 0 | 0 | 33 | 0 | 0 | 0 | 6 | 21 | 40 | 1 |
| 4 | 41 | LMNP | 0 | 0 | 0 | 0 | 46 | 14 | 0 | 0 | 9 | 1 | 13 | 0 |
| 5 | 226 | LMNP | 1 | 0 | 0 | 0 | 51 | 0 | 0 | 1 | 3 | 7 | 6 | 1 |
| 6 | 208 | LMNP | 0 | 0 | 0 | 0 | 67 | 1 | 0 | 0 | 9 | 4 | 27 | 1 |
| 7 | 195 | LMNP | 0 | 0 | 0 | 0 | 215 | 0 | 0 | 0 | 1 | 40 | 21 | 0 |
| 8 | 141 | LMNP | 4 | 0 | 0 | 0 | 62 | 2 | 0 | 0 | 18 | 0 | 1 | 4 |
| 9 | 226 | LMNP | 5 | 0 | 0 | 0 | 148 | 0 | 0 | 1 | 2 | 242 | 2 | 11 |
| 10 | 141 | LMNP | 4 | 0 | 0 | 0 | 152 | 0 | 0 | 0 | 59 | 8 | 0 | 4 |
| 12 | 135 | LMNP | 3 | 0 | 9 | 0 | 77 | 0 | 0 | 1 | 24 | 100 | 1 | 7 |
| 15 | 140 | LMNP | 0 | 0 | 134 | 0 | 171 | 0 | 0 | 2 | 9 | 43 | 0 | 28 |
| 16 | 141 | LMNP | 0 | 0 | 54 | 0 | 26 | 0 | 0 | 2 | 6 | 49 | 0 | 4 |
| 18 | 141 | LMNP | 1 | 0 | 0 | 0 | 19 | 0 | 0 | 0 | 0 | 1 | 1 | 0 |
| 20 | 175 | LMNP | 0 | 0 | 28 | 0 | 103 | 0 | 0 | 3 | 10 | 16 | 5 | 1 |
| 21 | 143 | LMNP | 0 | 0 | 13 | 0 | 0 | 0 | 0 | 0 | 0 | 5 | 0 | 2 |
| 24 | 118 | LMNP | 1 | 0 | 0 | 0 | 68 | 1 | 0 | 0 | 40 | 6 | 14 | 0 |
| 25 | 225 | LMNP | 1 | 1 | 9 | 0 | 67 | 0 | 0 | 1 | 3 | 36 | 7 | 1 |
| 26 | 141 | LMNP | 1 | 0 | 16 | 0 | 43 | 0 | 0 | 0 | 3 | 19 | 20 | 0 |
| 30 | 158 | LMNP | 1 | 0 | 4 | 0 | 9 | 0 | 0 | 1 | 1 | 1 | 12 | 6 |
| 31 | 141 | LMNP | 0 | 0 | 0 | 0 | 9 | 3 | 0 | 1 | 0 | 17 | 1 | 0 |
| 32 | 85 | LMNP | 0 | 0 | 5 | 0 | 3 | 0 | 0 | 1 | 5 | 12 | 0 | 0 |
| 36 | 80 | LMNP | 2 | 0 | 1 | 0 | 0 | 0 | 0 | 1 | 8 | 15 | 0 | 1 |
| 37 | 190 | LMNP | 0 | 0 | 32 | 0 | 7 | 0 | 0 | 0 | 2 | 8 | 0 | 0 |
| 38 | 71 | LMNP | 0 | 0 | 6 | 0 | 2 | 0 | 0 | 0 | 0 | 10 | 0 | 0 |
| 40 | 101 | LMNP | 3 | 0 | 14 | 0 | 8 | 0 | 0 | 1 | 3 | 2 | 0 | 5 |
| 43 | 306 | LMNP | 0 | 0 | 27 | 0 | 28 | 0 | 0 | 2 | 7 | 43 | 0 | 8 |
| 46 | 125 | LMNP | 5 | 0 | 5 | 0 | 29 | 0 | 0 | 0 | 4 | 29 | 0 | 0 |
| 47 | 160 | LMNP | 0 | 0 | 62 | 0 | 2 | 0 | 0 | 0 | 0 | 42 | 5 | 3 |
| 50 | 226 | LMNP | 2 | 0 | 1 | 0 | 21 | 0 | 0 | 3 | 3 | 2 | 0 | 1 |
| 51 | 96 | LMNP | 2 | 0 | 40 | 0 | 0 | 0 | 0 | 0 | 27 | 3 | 0 | 4 |
| 54 | 106 | LMNP | 4 | 0 | 7 | 0 | 26 | 0 | 0 | 2 | 7 | 15 | 0 | 0 |
| 55 | 140 | LMNP | 0 | 0 | 32 | 0 | 9 | 0 | 0 | 1 | 2 | 6 | 0 | 3 |
| 59 | 140 | LMNP | 0 | 0 | 17 | 0 | 7 | 0 | 0 | 0 | 3 | 10 | 0 | 0 |
| 63 | 84 | LMNP | 0 | 0 | 1 | 0 | 5 | 0 | 0 | 0 | 0 | 1 | 2 | 0 |
| 64 | 140 | LMNP | 0 | 0 | 11 | 0 | 0 | 0 | 0 | 0 | 3 | 7 | 0 | 4 |
| 70 | 132 | LMNP | 0 | 0 | 0 | 0 | 2 | 0 | 0 | 3 | 0 | 1 | 0 | 2 |
| 71 | 134 | LMNP | 0 | 0 | 210 | 0 | 35 | 0 | 1 | 0 | 4 | 55 | 0 | 4 |
| 77 | 92 | LMNP | 0 | 0 | 8 | 0 | 102 | 0 | 0 | 0 | 1 | 8 | 1 | 0 |
| 78 | 123 | LMNP | 0 | 0 | 0 | 0 | 157 | 0 | 0 | 0 | 0 | 239 | 0 | 3 |
| 82 | 140 | LMNP | 0 | 0 | 1 | 0 | 109 | 2 | 0 | 0 | 0 | 14 | 0 | 0 |
| 83 | 24 | LMNP | 1 | 0 | 0 | 0 | 0 | 0 | 0 | 0 | 11 | 0 | 0 | 0 |
| 87 | 101 | LMNP | 5 | 0 | 0 | 1 | 46 | 0 | 0 | 0 | 15 | 2 | 1 | 0 |
| 90 | 74 | LMNP | 0 | 0 | 0 | 0 | 12 | 0 | 0 | 0 | 0 | 0 | 2 | 0 |
| 91 | 111 | LMNP | 0 | 0 | 0 | 0 | 117 | 0 | 0 | 0 | 2 | 0 | 4 | 0 |
| Site 1 | 70 | Tlika | 0 | 0 | 0 | 0 | 0 | 0 | 0 | 0 | 2 | 0 | 0 | 0 |
| Site 11 | 140 | Tlika | 0 | 0 | 0 | 0 | 2 | 0 | 0 | 0 | 1 | 2 | 0 | 0 |
| Site 12 | 100 | Tlika | 0 | 0 | 11 | 0 | 1 | 0 | 0 | 0 | 3 | 18 | 0 | 0 |
| Site 13 | 69 | Tlika | 0 | 0 | 0 | 0 | 0 | 0 | 0 | 0 | 1 | 1 | 0 | 0 |
| Site 14 | 139 | Tlika | 0 | 0 | 0 | 0 | 0 | 0 | 0 | 2 | 2 | 3 | 0 | 1 |
| Site 15 | 139 | Tlika | 1 | 2 | 0 | 0 | 0 | 0 | 0 | 0 | 0 | 2 | 0 | 0 |
| Site 16 | 140 | Tlika | 0 | 1 | 4 | 0 | 3 | 0 | 0 | 0 | 1 | 3 | 0 | 0 |
| Site 17 | 139 | Tlika | 0 | 11 | 0 | 0 | 1 | 0 | 0 | 0 | 15 | 0 | 0 | 4 |
| Site 18 | 139 | Tlika | 0 | 0 | 0 | 0 | 0 | 0 | 0 | 3 | 2 | 2 | 0 | 0 |
| Site 19 | 89 | Tlika | 0 | 0 | 0 | 0 | 1 | 0 | 0 | 0 | 3 | 4 | 0 | 0 |
| Site 2 | 99 | Tlika | 0 | 0 | 0 | 0 | 0 | 0 | 0 | 0 | 1 | 0 | 0 | 0 |
| Site 20 | 81 | Tlika | 0 | 0 | 0 | 0 | 0 | 0 | 0 | 0 | 0 | 0 | 0 | 0 |
| Site 3 | 138 | Tlika | 0 | 10 | 0 | 0 | 2 | 0 | 0 | 0 | 0 | 0 | 0 | 0 |
| Site 4 | 138 | Tlika | 0 | 0 | 0 | 0 | 3 | 0 | 0 | 2 | 0 | 0 | 0 | 3 |
| Site 5 | 68 | Tlika | 0 | 0 | 0 | 0 | 0 | 0 | 0 | 0 | 5 | 0 | 0 | 0 |
| Site 6 | 139 | Tlika | 0 | 0 | 0 | 0 | 1 | 0 | 0 | 1 | 3 | 0 | 0 | 1 |
| Site 7 | 108 | Tlika | 0 | 0 | 0 | 0 | 0 | 0 | 0 | 3 | 0 | 2 | 0 | 0 |
| Site 8 | 139 | Tlika | 1 | 0 | 1 | 0 | 0 | 0 | 0 | 4 | 0 | 0 | 0 | 0 |
| Site 9 | 108 | Tlika | 5 | 0 | 0 | 0 | 1 | 0 | 0 | 0 | 3 | 17 | 0 | 7 |

| Station | Nights | Site | Wildebeest | Zebra | Zorilla | Wild dog | Bat-eared fox | Bush duiker | Caracal | Greater kudu | Klipspringer | Striped hyena | Wild cat | Eland | Cattle | Donkey | Shoat | Dog |
| --- | --- | --- | --- | --- | --- | --- | --- | --- | --- | --- | --- | --- | --- | --- | --- | --- | --- | --- |
| 0 | 141 | LMNP | 0 | 0 | 0 | 0 | 0 | 0 | 0 | 0 | 0 | 0 | 0 | 0 | 0 | 0 | 0 | 0 |
| 1 | 177 | LMNP | 155 | 41 | 2 | 0 | 0 | 0 | 0 | 0 | 0 | 0 | 0 | 0 | 0 | 0 | 0 | 0 |
| 2 | 156 | LMNP | 0 | 38 | 0 | 0 | 0 | 0 | 0 | 0 | 0 | 0 | 0 | 0 | 0 | 0 | 0 | 0 |
| 3 | 188 | LMNP | 4 | 20 | 0 | 0 | 0 | 0 | 0 | 0 | 0 | 0 | 0 | 0 | 0 | 0 | 0 | 0 |
| 4 | 41 | LMNP | 0 | 0 | 0 | 0 | 0 | 0 | 0 | 0 | 0 | 0 | 0 | 0 | 0 | 0 | 0 | 0 |
| 5 | 226 | LMNP | 2 | 12 | 2 | 0 | 0 | 0 | 0 | 0 | 0 | 0 | 0 | 0 | 0 | 0 | 0 | 0 |
| 6 | 208 | LMNP | 0 | 17 | 0 | 0 | 0 | 0 | 0 | 0 | 0 | 0 | 0 | 0 | 0 | 0 | 0 | 0 |
| 7 | 195 | LMNP | 12 | 18 | 0 | 0 | 0 | 0 | 0 | 0 | 0 | 0 | 0 | 0 | 0 | 0 | 0 | 0 |
| 8 | 141 | LMNP | 0 | 0 | 0 | 0 | 0 | 0 | 0 | 0 | 0 | 0 | 0 | 0 | 0 | 0 | 0 | 0 |
| 9 | 226 | LMNP | 770 | 56 | 0 | 0 | 0 | 0 | 0 | 0 | 0 | 0 | 0 | 0 | 0 | 0 | 0 | 0 |
| 10 | 141 | LMNP | 6 | 7 | 0 | 0 | 0 | 0 | 0 | 0 | 0 | 0 | 0 | 0 | 0 | 0 | 0 | 0 |
| 12 | 135 | LMNP | 3 | 3 | 0 | 0 | 0 | 0 | 0 | 0 | 0 | 0 | 0 | 0 | 0 | 0 | 0 | 0 |
| 15 | 140 | LMNP | 103 | 47 | 0 | 0 | 0 | 0 | 0 | 0 | 0 | 0 | 0 | 0 | 0 | 0 | 0 | 0 |
| 16 | 141 | LMNP | 126 | 119 | 1 | 0 | 0 | 0 | 0 | 0 | 0 | 0 | 0 | 0 | 0 | 0 | 0 | 0 |
| 18 | 141 | LMNP | 0 | 0 | 0 | 0 | 0 | 0 | 0 | 0 | 0 | 0 | 0 | 0 | 0 | 0 | 0 | 0 |
| 20 | 175 | LMNP | 2 | 4 | 0 | 0 | 0 | 0 | 0 | 0 | 0 | 0 | 0 | 0 | 0 | 0 | 0 | 0 |
| 21 | 143 | LMNP | 8 | 8 | 0 | 0 | 0 | 0 | 0 | 0 | 0 | 0 | 0 | 0 | 0 | 0 | 0 | 0 |
| 24 | 118 | LMNP | 0 | 0 | 0 | 0 | 0 | 0 | 0 | 0 | 0 | 0 | 0 | 0 | 0 | 0 | 0 | 0 |
| 25 | 225 | LMNP | 0 | 0 | 0 | 0 | 0 | 0 | 0 | 0 | 0 | 0 | 0 | 0 | 0 | 0 | 0 | 0 |
| 26 | 141 | LMNP | 0 | 0 | 0 | 0 | 0 | 0 | 0 | 0 | 0 | 0 | 0 | 0 | 0 | 0 | 0 | 0 |
| 30 | 158 | LMNP | 0 | 8 | 0 | 0 | 0 | 0 | 0 | 0 | 0 | 0 | 0 | 0 | 0 | 0 | 0 | 0 |
| 31 | 141 | LMNP | 0 | 0 | 0 | 0 | 0 | 0 | 0 | 0 | 0 | 0 | 0 | 0 | 0 | 0 | 0 | 0 |
| 32 | 85 | LMNP | 0 | 4 | 0 | 0 | 0 | 0 | 0 | 0 | 0 | 0 | 0 | 0 | 0 | 0 | 0 | 0 |
| 36 | 80 | LMNP | 0 | 3 | 0 | 0 | 0 | 0 | 0 | 0 | 0 | 0 | 0 | 0 | 0 | 0 | 0 | 0 |
| 37 | 190 | LMNP | 16 | 12 | 0 | 0 | 0 | 0 | 0 | 0 | 0 | 0 | 0 | 0 | 0 | 0 | 0 | 0 |
| 38 | 71 | LMNP | 0 | 0 | 0 | 0 | 0 | 0 | 0 | 0 | 0 | 0 | 0 | 0 | 0 | 0 | 0 | 0 |
| 40 | 101 | LMNP | 0 | 4 | 0 | 0 | 0 | 0 | 0 | 0 | 0 | 0 | 0 | 0 | 0 | 0 | 0 | 0 |
| 43 | 306 | LMNP | 0 | 8 | 0 | 0 | 0 | 0 | 0 | 0 | 0 | 0 | 0 | 0 | 0 | 0 | 0 | 0 |
| 46 | 125 | LMNP | 0 | 0 | 0 | 0 | 0 | 0 | 0 | 0 | 0 | 0 | 0 | 0 | 0 | 0 | 0 | 0 |
| 47 | 160 | LMNP | 60 | 158 | 0 | 0 | 0 | 0 | 0 | 0 | 0 | 0 | 0 | 0 | 0 | 0 | 0 | 0 |
| 50 | 226 | LMNP | 0 | 0 | 0 | 0 | 0 | 0 | 0 | 0 | 0 | 0 | 0 | 0 | 0 | 0 | 0 | 0 |
| 51 | 96 | LMNP | 123 | 167 | 0 | 0 | 0 | 0 | 0 | 0 | 0 | 0 | 0 | 0 | 0 | 0 | 0 | 0 |
| 54 | 106 | LMNP | 0 | 3 | 0 | 0 | 0 | 0 | 0 | 0 | 0 | 0 | 0 | 0 | 0 | 0 | 0 | 0 |
| 55 | 140 | LMNP | 4 | 11 | 0 | 0 | 0 | 0 | 0 | 0 | 0 | 0 | 0 | 0 | 0 | 0 | 0 | 0 |
| 59 | 140 | LMNP | 0 | 30 | 0 | 0 | 0 | 0 | 0 | 0 | 0 | 0 | 0 | 0 | 0 | 0 | 0 | 0 |
| 63 | 84 | LMNP | 0 | 0 | 0 | 0 | 0 | 0 | 0 | 0 | 0 | 0 | 0 | 0 | 0 | 0 | 0 | 0 |
| 64 | 140 | LMNP | 0 | 17 | 0 | 0 | 0 | 0 | 0 | 0 | 0 | 0 | 0 | 0 | 0 | 0 | 0 | 0 |
| 70 | 132 | LMNP | 0 | 3 | 0 | 0 | 0 | 0 | 0 | 0 | 0 | 0 | 0 | 0 | 0 | 0 | 0 | 0 |
| 71 | 134 | LMNP | 307 | 172 | 0 | 0 | 0 | 0 | 0 | 0 | 0 | 0 | 0 | 0 | 0 | 0 | 0 | 0 |
| 77 | 92 | LMNP | 0 | 3 | 0 | 0 | 0 | 0 | 0 | 0 | 0 | 0 | 0 | 0 | 0 | 0 | 0 | 0 |
| 78 | 123 | LMNP | 47 | 44 | 0 | 0 | 0 | 0 | 0 | 0 | 0 | 0 | 0 | 0 | 0 | 0 | 0 | 0 |
| 82 | 140 | LMNP | 1 | 0 | 0 | 0 | 0 | 0 | 0 | 0 | 0 | 0 | 0 | 0 | 0 | 0 | 0 | 0 |
| 83 | 24 | LMNP | 0 | 0 | 0 | 0 | 0 | 0 | 0 | 0 | 0 | 0 | 0 | 0 | 0 | 0 | 0 | 0 |
| 87 | 101 | LMNP | 0 | 0 | 0 | 0 | 0 | 0 | 0 | 0 | 0 | 0 | 0 | 0 | 0 | 0 | 0 | 0 |
| 90 | 74 | LMNP | 0 | 0 | 0 | 0 | 0 | 0 | 0 | 0 | 0 | 0 | 0 | 0 | 0 | 0 | 0 | 0 |
| 91 | 111 | LMNP | 0 | 0 | 0 | 0 | 0 | 0 | 0 | 0 | 0 | 0 | 0 | 0 | 0 | 0 | 0 | 0 |
| Site 1 | 70 | Tlika | 0 | 0 | 0 | 0 | 0 | 0 | 0 | 0 | 0 | 0 | 0 | 1 | 25 | 3 | 2 | 1 |
| Site 11 | 140 | Tlika | 0 | 0 | 0 | 0 | 0 | 0 | 2 | 2 | 0 | 0 | 0 | 0 | 20 | 0 | 0 | 0 |
| Site 12 | 100 | Tlika | 0 | 0 | 0 | 0 | 0 | 1 | 0 | 31 | 3 | 0 | 2 | 0 | 9 | 1 | 0 | 3 |
| Site 13 | 69 | Tlika | 0 | 0 | 0 | 0 | 0 | 5 | 0 | 0 | 0 | 0 | 0 | 0 | 8 | 2 | 0 | 0 |
| Site 14 | 139 | Tlika | 0 | 0 | 0 | 0 | 0 | 0 | 0 | 1 | 0 | 0 | 1 | 0 | 3 | 0 | 0 | 0 |
| Site 15 | 139 | Tlika | 0 | 0 | 0 | 0 | 0 | 1 | 0 | 1 | 0 | 0 | 0 | 0 | 20 | 2 | 2 | 1 |
| Site 16 | 140 | Tlika | 0 | 0 | 0 | 0 | 0 | 4 | 0 | 23 | 0 | 2 | 0 | 0 | 10 | 0 | 0 | 1 |
| Site 17 | 139 | Tlika | 0 | 0 | 0 | 0 | 2 | 1 | 0 | 2 | 0 | 0 | 0 | 0 | 20 | 16 | 0 | 38 |
| Site 18 | 139 | Tlika | 0 | 0 | 0 | 0 | 0 | 1 | 0 | 11 | 0 | 0 | 0 | 0 | 2 | 0 | 0 | 0 |
| Site 19 | 89 | Tlika | 0 | 0 | 0 | 0 | 0 | 1 | 0 | 2 | 0 | 0 | 0 | 0 | 16 | 2 | 1 | 1 |
| Site 2 | 99 | Tlika | 0 | 0 | 0 | 0 | 0 | 0 | 0 | 1 | 0 | 1 | 0 | 0 | 9 | 0 | 0 | 0 |
| Site 20 | 81 | Tlika | 0 | 0 | 0 | 0 | 0 | 0 | 0 | 6 | 2 | 0 | 0 | 0 | 2 | 1 | 0 | 0 |
| Site 3 | 138 | Tlika | 0 | 0 | 0 | 0 | 0 | 1 | 0 | 10 | 0 | 0 | 0 | 0 | 3 | 0 | 0 | 0 |
| Site 4 | 138 | Tlika | 0 | 0 | 0 | 0 | 0 | 0 | 3 | 0 | 6 | 1 | 0 | 0 | 2 | 0 | 0 | 0 |
| Site 5 | 68 | Tlika | 0 | 0 | 0 | 0 | 0 | 0 | 0 | 0 | 0 | 1 | 0 | 0 | 7 | 0 | 0 | 0 |
| Site 6 | 139 | Tlika | 0 | 0 | 0 | 0 | 5 | 1 | 3 | 4 | 0 | 1 | 1 | 0 | 21 | 1 | 0 | 1 |
| Site 7 | 108 | Tlika | 0 | 0 | 0 | 0 | 0 | 0 | 0 | 0 | 0 | 0 | 0 | 0 | 0 | 0 | 0 | 0 |
| Site 8 | 139 | Tlika | 0 | 1 | 0 | 1 | 3 | 0 | 0 | 6 | 0 | 1 | 0 | 0 | 2 | 0 | 0 | 0 |
| Site 9 | 108 | Tlika | 0 | 0 | 0 | 0 | 1 | 0 | 0 | 2 | 0 | 1 | 0 | 0 | 1 | 0 | 0 | 0 |
