# Supplementary material for: Hunter-Gatherers in context: Mammal community composition in a northern Tanzania landscape used by Hadza foragers and Datoga pastoralists
Source: PLoS One. 2021 May 14;16(5):e0251076. doi: 10.1371/journal.pone.0251076 (PMC8121365; doi:10.1371/journal.pone.0251076)
Supplement: S2 Data — The associated p-value is based on a likelihood ratio test, comparing a model with site effect (LMNP vs. Tli’ika) with an intercept only model. (DOCX) [file pone.0251076.s003.docx]

**S2 Data. Mean of relative abundance indices (RAI; independent camera trap events / 100 camera trap nights) of mammal species in Lake Manyara National Park (LMNP) and the Kideru ridge (Tli’ika) of northern Tanzania.** The associated p-value is based on a likelihood ratio test, comparing a model with site effect (LMNP vs. Tli’ika) with an intercept only model.

| Species | Mean RAI (LMNP) | Mean RAI (Tli‘ika) | Δ RAI  (LMNP-Tli‘ika) | Likelihood ratio test statistic | P-value |
| --- | --- | --- | --- | --- | --- |
| Aardvark | 0.393 | 0.076 | 0.317 | 2.282 | 0.131 |
| Aardwolf | 0.012 | 0.276 | -0.264 | 10.938 | < 0.001 |
| Baboon | 36.331 | 0.615 | 35.716 | 45.626 | < 0.001 |
| Banded mongoose | 4.698 | 0 | 4.698 | 28.723 | < 0.001 |
| Bat-eared fox | 0.000 | 0.427 | -0.427 | 7.782 | 0.005 |
| Black-backed jackal | 2.223 | 1.988 | 0.235 | 0.023 | 0.880 |
| Buffalo | 19.488 | 0 | 19.488 | 45.754 | < 0.001 |
| Bush duiker | 0.000 | 0.833 | -0.833 | 21.152 | < 0.001 |
| Bush hyrax | 1.250 | 1.220 | 0.030 | 2.683 | 0.101 |
| Bushbuck | 10.984 | 2.206 | 8.779 | 11.409 | < 0.001 |
| Bushpig | 0.098 | 1.274 | -1.176 | 24.141 | < 0.001 |
| Bushy-tailed mongoose | 2.736 | 0.836 | 1.900 | 2.884 | 0.089 |
| Caracal | 0 | 0.303 | -0.303 | 5.661 | 0.017 |
| Cattle | 0 | 9.002 | -9.002 | 68.554 | < 0.001 |
| Civet | 0.288 | 0.896 | -0.608 | 2.362 | 0.124 |
| Common genet | 2.291 | 4.649 | -2.358 | 2.925 | 0.087 |
| Dog | 0 | 1.844 | -1.844 | 15.554 | < 0.001 |
| Donkey | 0 | 1.333 | -1.333 | 18.826 | < 0.001 |
| Dwarf mongoose | 0.697 | 0.190 | 0.506 | 2.961 | 0.085 |
| Egyptian mongoose | 0.035 | 0 | 0.035 | 0 | 0.985 |
| Eland | 0 | 0.075 | -0.075 | 0.565 | 0.452 |
| Elephant | 17.124 | 0.165 | 16.958 | 56.678 | < 0.001 |
| Giraffe | 11.760 | 0.767 | 10.993 | 9.742 | 0.002 |
| Greater galago | 0.216 | 0 | 0.216 | 2.016 | 0.156 |
| Greater kudu | 0 | 4.558 | -4.558 | 43.003 | < 0.001 |
| Hare | 0.354 | 1.280 | -0.926 | 3.226 | 0.072 |
| Hippopotamus | 8.543 | 0.000 | 8.543 | 13.250 | < 0.001 |
| Honey badger | 0.586 | 0.320 | 0.266 | 0.963 | 0.326 |
| Impala | 31.847 | 4.002 | 27.845 | 17.087 | < 0.001 |
| Kirk's dik-dik | 7.367 | 20.338 | -12.971 | 3.518 | 0.061 |
| Klipspringer | 0 | 0.517 | -0.517 | 5.870 | 0.015 |
| Large spotted genet | 1.062 | 1.323 | -0.260 | 0.210 | 0.647 |
| Leopard | 0.894 | 0.319 | 0.575 | 2.594 | 0.107 |
| Lesser galago | 0.010 | 0.911 | -0.902 | 9.190 | 0.002 |
| Lion | 0.767 | 0 | 0.767 | 7.521 | 0.006 |
| Manyara monkey | 0.994 | 0 | 0.994 | 3.628 | 0.057 |
| Marsh mongoose | 0.022 | 0 | 0.022 | 0.565 | 0.452 |
| Porcupine | 3.463 | 0.742 | 2.721 | 6.655 | 0.010 |
| Red duiker | 1.419 | 0 | 1.419 | 3.200 | 0.074 |
| Serval | 0.016 | 0 | 0.016 | 0.565 | 0.452 |
| Shoat | 0 | 0.285 | -0.285 | 5.663 | 0.017 |
| Slender mongoose | 0.412 | 0.601 | -0.189 | 1.083 | 0.298 |
| Spotted hyena | 6.736 | 2.057 | 4.680 | 6.743 | 0.009 |
| Striped hyena | 0 | 0.368 | -0.368 | 15.805 | < 0.001 |
| Vervet monkey | 17.599 | 2.639 | 14.960 | 14.108 | < 0.001 |
| Warthog | 9.715 | 0.406 | 9.308 | 36.699 | < 0.001 |
| Waterbuck | 4.087 | 0 | 4.087 | 17.609 | < 0.001 |
| White-tailed mongoose | 1.659 | 0.683 | 0.976 | 2.719 | 0.099 |
| Wild cat | 0 | 0.181 | -0.181 | 4.931 | 0.026 |
| Wild dog | 0 | 0.038 | -0.038 | 0.565 | 0.452 |
| Wildebeest | 23.011 | 0 | 23.011 | 12.593 | < 0.001 |
| Zebra | 16.342 | 0.038 | 16.304 | 26.193 | < 0.001 |
| Zorilla | 0.059 | 0 | 0.059 | 0.135 | 0.714 |
